# The Association Analysis of *GPNMB* rs156429 With Clinical Manifestations in Chinese Population With Parkinson's Disease

**DOI:** 10.3389/fgene.2020.00952

**Published:** 2020-09-02

**Authors:** Jin Liu, Gen Li, Yixi He, Guiying He, Pingchen Zhang, Xin Shen, Weishan Zhang, Shengdi Chen, Shishuang Cui, Yuyan Tan

**Affiliations:** ^1^Department of Neurology & Collaborative Innovation Center for Brain Science, Ruijin Hospital Affiliated to Shanghai Jiao Tong University School of Medicine, Shanghai, China; ^2^Department of Geriatrics, Ruijin Hospital Affiliated to Shanghai Jiao Tong University School of Medicine, Shanghai, China

**Keywords:** Parkinson's disease, single nucleotide polymorphisms, GPNMB, genetics, clinical manifestation

## Abstract

**Background:** The mechanisms of Parkinson's disease (PD) include complicated genetic factors. The roles of newly found risk genes need to be further verified among different ethnicities. In a two-stage meta-analysis, single nucleotide polymorphism (SNP) of rs156429 in glycoprotein non-metastatic melanoma protein B (*GPNMB*) was reported to be associated with PD. So far clinical studies have focused on association between rs156429 and PD onset, however there is little evidence linking rs156429 with PD symptoms.

**Objective:** This study aimed to investigate the possible association of *GPNMB* rs156429 with PD manifestations among southeastern Chinese people.

**Methods:** Demographic variables, disease-related factors, and motor and non-motor assessments of 511 PD patients were collected. Polymerase chain reaction (PCR) and SNaPshot technique were used to detect *GPNMB* rs156429. The associations of rs156429 with PD rating scales and clinical manifestations were analyzed by Kruskal-Wallis test and logistic regression model separately.

**Results:** Kruskal-Wallis test and logistic regression model failed to reveal an association between *GPNMB* rs156429 and scores from Montreal Cognitive Assessment (MoCA) (*p* = 0.037; *p* = 1.000 after correction), and pain symptoms of 511 PD patients (*p* = 0.008, OR = 0.59, 95% CI = 0.40–0.87, overdominant model after adjustment; *p* = 0.168 after correction, overdominant model after adjustment). However, further analysis based on genders showed that *GPNMB* rs156429 might have a trend for being associated with cognitive dysfunction (Mini-Mental State Examination (MMSE), *p* = 0.064 after correction; MoCA, *p* = 0.064 after correction) and pain symptoms (*p* = 0.063 after correction, overdominant model after adjustment) in female PD patients but not male patients.

**Conclusions:** This study revealed that *GPNMB* rs156429 might have a trend for being associated with cognitive dysfunction and pain symptoms of female PD patients in the southeastern Chinese population. Further studies from a larger sample size are needed to confirm these findings.

## Introduction

Parkinson's disease (PD) is one of the most common neurodegenerative disorders (Saeed et al., 2017). It is predominantly characterized by motor symptoms including bradykinesia, resting tremor, rigidity, and posture instability. In addition, non-motor manifestations like rapid eye movement sleep behavior disorder (RBD), hyposmia, depression, and autonomic dysfunction, are also prevalent in PD patients (Saeed et al., 2017). The mechanisms of PD are complex. Besides aging and neurotoxins, genetic factors are quite crucial for disease onset (Pang et al., 2019).

By now, several classic PD-associated loci like *SNCA, GBA, LRRK2, PARK7*, and *PINK1* have been broadly verified by both basic and clinical studies (Pang et al., 2017). With the application of genome-wide association studies (GWAS), many new loci have been identified (Do et al., 2011; Nalls et al., 2011, 2014). A two-stage meta-analysis found that glycoprotein non-metastatic melanoma protein B (*GPNMB*)/7p15 was associated with PD onset (International Parkinson's Disease Genomics Consortium (IPDGC) Wellcome Trust Case Control Consortium 2 (WTCCC2)., 2011). Several subsequent studies indicated the relationship between single nucleotide polymorphism (SNP) of *GPNMB* rs156429 and PD, in both the Scandinavian population (Pihlstrom et al., 2013) and the central Chinese population (Liu et al., 2015). In Liu's report, there were significant differences between male Chinese PD participants and healthy controls in both genotype (*p* = 0.01) and allele distribution (*p* = 0.01, OR = 0.67) of rs156429, indicating that *GPNMB* rs156429 could have a protective role in male Chinese patients with PD (Liu et al., 2015). However, results were inconsistent in other European-descended populations (Hernandez et al., 2012; Soto-Ortolaza et al., 2013; Kara et al., 2014) and the southwestern Chinese population (Xu et al., 2016), which failed to find the association of *GPNMB* rs156429 with PD. More related clinical studies with larger sample sizes are urgently needed. In despite of contradictory clinical results, researchers found that GPNMB was both transcriptionally and expressively elevated in the substantia nigra areas of PD patients (Murthy et al., 2017; Moloney et al., 2018; Neal et al., 2018). GPNMB could involve in regulation of inflammation and immune systems (Budge et al., 2018), which also participated in PD pathogenesis. In addition, one study found that overexpression of GPNMB could protect against neurodegeneration changes induced by neurotoxins (Budge et al., 2020), indicating that GPNMB could serve as an emerging target for neurodegenerative diseases (Budge et al., 2018).

As for association between *GPNMB* rs156429 and PD symptoms, there were few related findings. The results of one study conducted among elderly American people with mild Parkinsonian signs showed that this SNP polymorphism was associated with the number of steps taken in a timed 2.4-m gait trial (Shulman et al., 2014). Another study showed that rs156429 had no association with anxiety and depression symptoms in southwestern Chinese PD patients (Xu et al., 2016). To date, there has been no study focusing on the relationship between this SNP and detailed motor and non-motor symptoms of PD. Therefore, in this study, we aimed to investigate the association of *GPNMB* rs156429 with clinical manifestations of PD in the southeastern Chinese population, providing clinical evidences for exploring potential molecular mechanisms of *GPNMB* rs156429 function in PD.

## Methods

### Study Population

PD was diagnosed by movement disorder physicians according to diagnostic criteria established by movement disorders society (MDS) (Postuma et al., 2015). A total of 511 PD patients were recruited from 2016 to 2018 at Ruijin Hospital. Participants with atypical parkinsonism, secondary parkinsonism, and other movement disorders were excluded from this study. In addition, patients with comorbidities that might affect the reliable completion of clinical assessments were also excluded, such as severe visual or hearing impairment, and inability to write or speak. All participants in the study were fully informed, and signed consent forms. This study was approved by the ethics committee of Ruijin Hospital affiliated to Shanghai Jiao Tong University School of Medicine.

### Assessments

Clinical assessments were completed, and blood samples were collected. Demographic information includes age, gender, education level, and family history. We also analyzed the composition of PD subtypes as late-onset PD (LOPD) or early-onset PD (EOPD). Based on previous reports, LOPD was defined as the PD patient older than 45 years old at the time of first diagnosis (González-Del Rincón et al., 2013). Clinical assessments include Hoehn-Yahr (H-Y) staging; the Movement Disorder Society Unified Parkinson's Disease Rating Scale (MDS-UPDRS) (Martinez-Martin et al., 2015); Non-Motor Symptoms Scale (NMSS) (Martinez-Martin et al., 2009); 39-item Parkinson's disease Questionnaire (PDQ-39) (Galeoto et al., 2018); Sniffin' Sticks-16 (SS-16) (Chen et al., 2015); Hamilton Anxiety Rating Scale (HAMA) (Kummer et al., 2010); Hamilton Depression Rating Scale (HAMD) (Kummer et al., 2010); Brief Pain Inventory (BPI) (Lin et al., 2016); Rapid Eye Movement Sleep Behavior Disorder Questionnaire-Hong Kong Version (RBD-HK) (Shen et al., 2014); Parkinson's disease Sleep Scale (PDSS) (Selvaraj and Keshavamurthy, 2016); Fatigue Severity Scale (FSS) (Fu et al., 2017); Epworth Sleeping Scale (ESS) (Ataide et al., 2014); Scales for Outcomes in Parkinson's disease-Autonomic Questionnaire (SCOPA-AUT) (Visser et al., 2004); Mini-Mental State Examination (MMSE); and Montreal Cognitive Assessment (MoCA) Beijing Version (Hoops et al., 2009). Disease-related changes in non-motor function, activity of daily living (ADL), and motor function were assessed with MDS-UPDRS parts 1, 2, and 3. General life quality was assessed by PDQ-39. The overall non-motor burden was measured by NMSS. Depression and anxiety levels were assessed by HAMD and HAMA, respectively. Pain symptoms were measured by BPI. Sleep disturbance was assessed by PDSS. Fatigue was measured by FSS, and somnolence was measured by ESS. Autonomic function was assessed by SCOPA-AUT. Cognitive status was assessed by MMSE and MoCA Beijing Version. Olfactory function was assessed by SS-16, and hyposmia was determined when the score of SS-16 was <8.3 points (Chen et al., 2015). RBD was assessed by RBD-HK, and probable RBD was diagnosed according to previous literature (Shen et al., 2014). Other clinical symptoms, such as dysphagia, sialorrhea, constipation, nocturia, etc., as shown in Supplementary Table 2, were defined as present or absent by two other movement disorder physicians (not those who did the scale assessments).

### DNA Preparations and Genotyping

Peripheral blood samples were collected, and DNA was extracted from leukocytes by using phenol–chloroform isopropyl alcohol method. Primers were designed by Primer 5 (version 5.00, PREMIER Biosoft International). After purification of polymerase chain reaction (PCR) products by both phosphorylase (FastAP, Applied biosystems) and exonuclease I (EXO I, Applied biosystems), extension reaction was done by SNaPshot Multiplex kit of ABI. The extended products were further purified by phosphorylase (FastAP, Applied biosystems), and sampled by ABI3730xl (Applied biosystems). The results of SNP typing were analyzed by genemap 4.0 (Applied biosystems).

The primer information is as follows:

Forward: TCC TGG GGT GTT TGA ATC ATA AG

Reverse: TGT GTT CAC ACA AAA TGT GGG ATT A

Elongation: TGC TTT TTA GAA AAA TAT CAG GAA C.

### Statistical Analysis

Both R (version 3.5.1) and RStudio (version 1.1.463, RStudio, Inc.) were applied for statistical analysis. The packages used included readxl package (version 1.2.0), CATT package (version 2.0), base package (version 3.5.1), and stats package (version 3.5.1) (package link: https://cran.r-project.org/web/packages/index.html). Chi-square test or Fisher exact test was adopted to assess Hardy-Weinberg equilibrium (HWE), and compare the differences in gender, PD subtype, education level, Hoehn-Yahr staging, and family history among *GPNMB* rs156429 subgroups. ANOVA was applied to test the differences of age at interview. For continuous outcomes (such as MMSE, MoCA, BPI, HAMD, etc.), we first checked whether those data followed the normal distribution. If so, ANOVA was applied. Otherwise, Kruskal-Wallis test was applied for non-parametric variables. Some PD symptoms, such as hyposmia, probable RBD, etc., were recorded as categorical variables. To investigate the association between *GPNMB* rs156429 and those symptoms, logistic regression model was adopted, and four genetic models (additive model, dominant model, recessive model, and overdominant model) were analyzed. These models were all further adjusted by Hoehn-Yahr staging and gender. Odds ratio (OR), 95% confidence interval (CI), and *p*-value (two-tailed test) were computed. The *p*-values were further assessed by Bonferroni correction of multiple tests (Bonferroni correction of multiple tests was conducted in 32 rating scales and sub-scales, such as SS-16, HAMA, HAMD, MDS-UPDRS parts 1, 2, and 3, etc.; and 21 symptoms, such as dysphagia, sialorrhea, constipation, nocturia, etc).

## Results

A total of 511 PD participants were recruited in this study. There were 280 male PD patients (54.79%) and 231 female patients (45.21%). Three hundred and four of total recruited PD patients were TT genotype (59.49%), 176 were CT genotype (34.44%) and 31 were CC genotype (6.07%). *GPNMB* rs156429 was tested in HWE (*p* = 0.851). There was no statistical difference in gender, age at interview, PD subtype, education level, Hoehn-Yahr staging, and family history in the three genotypes of PD patients (Table 1).

**Table 1:** Demographic information of *GPNMB* rs156429 in PD patients.

	**TT genotype (*n* = 304)**	**CT genotype (*n* = 176)**	**CC genotype (*n* = 31)**	***p*-value**
Gender (female, *n*, %)	133 (43.75)	80 (45.45)	18 (58.06)	0.790
Age at interview (mean ± SD)	62.24 (10.47)	63.92 (11.37)	63.86 (9.11)	0.115
Subtype (LOPD, *n*, %)	280 (92.11)	165 (93.75)	30 (96.77)	0.657
Education (*n*, %)				0.638
Illiterate	11 (3.73)	2 (1.18)	1 (3.45)	
≤6 years	45 (15.25)	27 (15.88)	6 (20.69)	
6–12 years	158 (53.56)	93 (54.71)	17 (58.62)	
>12 years	81 (27.46)	48 (28.24)	5 (17.24)	
Hoehn-Yahr staging (*n*, %)				0.423
1	80 (28.99)	40 (25.00)	8 (27.59)	
1.5	44 (15.94)	24 (15.00)	5 (17.24)	
2	119 (43.12)	78 (48.75)	10 (34.48)	
2.5	12 (4.35)	6 (3.75)	3 (10.34)	
3	14 (5.07)	10 (6.25)	1 (3.45)	
4	6 (2.17)	0 (0.00)	1 (3.45)	
5	1 (0.36)	2 (1.25)	1 (3.45)	
Family history (*n*, %)	31 (10.20)	26 (14.94)	4 (12.90)	0.303

*LOPD, late-onset Parkinson's disease; SD, standard deviation*.

The detailed PD rating scale information in all three genotypes of rs156429 was analyzed (Supplementary Table 1). All scores did not follow the normal distribution, thus Kruskal-Wallis test was applied to analyze the association of rating scales scores and *GPNMB* rs156429 genotypes. There was no significant association between *GPNMB* rs156429 and PD rating scales (*p* = 0.037, MoCA; *p* = 1.000 after correction, MoCA) (Table 2). Multiple pairwise-comparison was further applied to investigate the differences of rating scales among genotypes of *GPNMB* rs156429. Similarly, MoCA scores failed to pass Bonferroni correction between TT and CC genotypes of PD patients (*p* = 0.034, MoCA; *p* = 1.000 after correction, MoCA).

**Table 2:** The association between PD rating scales and genotype of *GPNMB* rs156429 in PD patients.

	***p*-value**	***p*-value^a^**	***p*****-value (multiple pairwise-comparison between groups)**		
	**TT vs. CT vs. CC (*n* = 304 vs. 176 vs. 31)**	**TT vs. CT vs. CC (*n* = 304 vs. 176 vs. 31)**	**TT vs. CT (*n* = 304 vs. 176)**	**TT vs. CC (*n* = 304 vs. 31)**	**CT vs. CC (*n* = 176 vs. 31)**
SS-16	0.277	1.000	0.343	0.343	0.544
HAMA	0.625	1.000	0.725	0.595	0.595
HAMD	0.690	1.000	0.884	0.643	0.643
BPI	0.200	1.000	0.321	0.321	0.602
RBD-HK	0.848	1.000	0.838	0.838	0.838
PDSS	0.643	1.000	0.925	0.925	0.962
PDQ39	0.441	1.000	0.618	0.815	0.815
FSS	0.738	1.000	0.910	0.910	0.949
ESS	0.538	1.000	0.968	0.473	0.473
MDS-UPDRS	0.649	1.000	0.641	0.641	0.641
PART I	0.929	1.000	0.973	0.973	0.973
PART II	0.561	1.000	0.590	0.590	0.590
PART III	0.792	1.000	0.745	0.745	0.745
NMSS	0.804	1.000	0.830	0.830	0.830
cardiovascular	0.876	1.000	0.770	0.770	0.770
sleep	0.623	1.000	0.688	0.688	0.704
mood disorder	0.993	1.000	0.983	0.983	0.983
delusion	0.884	1.000	0.982	0.946	0.946
attention	0.785	1.000	0.965	0.775	0.775
gastrointestinal	0.526	1.000	0.544	0.544	0.544
urinary	0.920	1.000	0.918	0.918	0.918
sexual dysfunction	0.737	1.000	0.908	0.908	0.908
others	0.749	1.000	0.805	0.801	0.801
SCOPA-AUT	0.944	1.000	0.978	0.978	0.978
gastrointestinal	0.851	1.000	0.792	0.792	0.792
urinary	0.973	1.000	0.927	0.927	0.927
cardiovascular	0.908	1.000	0.847	0.847	0.847
skin	0.648	1.000	0.705	0.663	0.663
sexual dysfunction	0.942	1.000	0.898	0.898	0.898
drug usage	0.536	1.000	0.569	0.569	0.569
MMSE	0.059	1.000	0.516	0.055	0.055
MoCA	**0.037**	1.000	0.392	**0.034**	0.062

*BPI, brief pain inventory; ESS, Epworth Sleepiness Scale; FSS, Fatigue severity scale; HAMA, Hamilton anxiety rating scale; HAMD, Hamilton depression rating scale; MDS, movement disorders society; MMSE, Mini-Mental State Examination; MoCA, Montreal Cognitive Assessment; NMSS, Non-Motor Symptoms Scale; PDQ-39, 39-item Parkinson's Disease Questionnaire; PDSS, Parkinson's disease sleep scale; RBD-HK, rapid eye movement sleep behavior disorder questionnaire-Hong Kong version; SCOPA-AUT, Scales for Outcomes in Parkinson's Disease-Autonomic questionnaire; SS-16, Sniffin' Sticks 16; UPDRS, Unified Parkinson's Disease Rating Scale*.

a *p-value after Bonferroni correction*.

*Bold fonts: p < 0.05*.

Four genetic models (additive model, dominant model, recessive model, and overdominant model) were applied to investigate the association between PD symptoms and *GPNMB* rs156429 polymorphism (Supplementary Table 2). Pain symptoms were shown to have a trend for being associated with *GPNMB* rs156429 in dominant model (*p* = 0.021, OR = 1.56, 95% CI = 1.07–2.28, dominant model after adjustment; *p* = 0.441 after correction, dominant model after adjustment) and overdominant model (*p* = 0.008, OR = 0.59, 95% CI = 0.40–0.87, overdominant model after adjustment; *p* = 0.168 after correction, overdominant model after adjustment) (Table 3, Supplementary Table 2). The inheritance pattern of *GPNMB* rs156429 is not clear, and determination of inheritance pattern that a trait will follow is still unknown. Thus, in different genetic models, the genotype might show different or even opposite ORs. Considering the small sample size of monocentric PD participants in our study, the association between pain and *GPNMB* rs156429 in PD was still highly expected in future studies with more participants.

**Table 3:** The association between pain symptoms and genetic models of *GPNMB* rs156429 in PD patients.

	**Genetic model**							**Genetic model with adjustment**						
	**Dominant model**							**Dominant model (adjusted)**^a^						
	**Beta**	**SE**	**Power**	***p*****-value**	**OR**	**95% CI**	***p*****-value**^c^	**Beta**	**SE**	**Power**	***p*****-value**	**OR**	**95% CI**	***p*****-value**^c^
	0.417	0.183	0.132	**0.023**	**1.52**	**(1.06, 2.18)**	0.483	0.444	0.193	0.136	**0.021**	**1.56**	**(1.07, 2.28)**	0.441
**PD patients (*****n*** **= 511)**	**Overdominant model**							**Overdominant model (adjusted)**^a^						
	**Beta**	**SE**	**Power**	***p*****-value**	**OR**	**95% CI**	***p*****-value**^c^	**Beta**	**SE**	**Power**	***p*****-value**	**OR**	**95% CI**	***p*****-value**^c^
	−0.473	0.189	0.100	**0.012**	**0.62**	**(0.43, 0.90)**	0.252	−0.531	0.199	0.095	**0.008**	**0.59**	**(0.40, 0.87)**	0.168
	**Dominant model**							**Dominant model (adjusted)**^b^						
	**Beta**	**SE**	**power**	***p*****-value**	**OR**	**95% CI**	***p*****-value**^c^	**beta**	**SE**	**power**	***p*****-value**	**OR**	**95% CI**	***p*****-value**^c^
	0.684	0.272	0.115	**0.012**	**1.98**	**(1.17, 3.39)**	0.252	0.687	0.285	0.133	**0.016**	**1.99**	**(1.14, 3.49)**	0.336
**Female PD patients (*****n*** **= 231)**	**Overdominant model**							**Overdominant model (adjusted)**^b^						
	**Beta**	**SE**	**Power**	***p*****-value**	**OR**	**95% CI**	***p*****-value**^c^	**Beta**	**SE**	**Power**	***p*****-value**	**OR**	**95% CI**	***p*****-value**^c^
	−0.835	0.285	0.093	**0.003**	**0.43**	**(0.25, 0.75)**	0.063	−0.898	0.301	0.095	**0.003**	**0.41**	**(0.22, 0.73)**	0.063

*CI, confidence interval; OR, odds ratio; SE, standard error*.

a *Hoehn-Yahr staging and gender were taken as adjustments*.

b *Hoehn-Yahr staging was taken as adjustment*.

c *p-value after Bonferroni correction*.

*Bold fonts: p < 0.05*.

Previous literature reported that *GPNMB* rs156429 could have a protective role in male Chinese patients with PD (Liu et al., 2015). Therefore, we analyzed data in male and female patients separately. Both MMSE and MoCA had a trend in association analysis with *GPNMB* rs156429 genotypes in female PD patients (*p* = 0.002, MMSE; *p* = 0.064 after correction, MMSE; *p* = 0.002, MoCA; *p* = 0.064 after correction, MoCA) (Supplementary Table 3), so did pain symptoms in association analysis with rs156429 in female PD patients (*p* = 0.016, OR = 1.99, 95% CI = 1.14–3.49, dominant model after adjustment; *p* = 0.336 after correction, dominant model after adjustment; *p* = 0.003, OR = 0.41, 95% CI = 0.22–0.73, overdominant model after adjustment; *p* = 0.063 after correction, overdominant model after adjustment) (Table 3, Supplementary Table 4). For male PD patients, no statistical differences in PD symptoms were found among three rs156429 genotypes (Supplementary Table 5), or in four genetic models (Supplementary Table 6).

## Discussion

In this study, we revealed for the first time that *GPNMB* rs156429 polymorphism might have a trend for being associated with cognitive dysfunction and pain symptoms among southeastern female Chinese PD patients but not male patients. These findings could provide evidence that *GPNMB* rs156429 might have different effects on PD symptoms between female and male patients.

*GPNMB* was identified as one of the newly found risk loci of PD in a two-stage meta-analysis study, and SNP rs156429 was considered to be the most relevant candidate for PD [International Parkinson's Disease Genomics Consortium (IPDGC) and Wellcome Trust Case Control Consortium 2 (WTCCC2), 2011]. There is minimal supporting literature referring to the association of *GPNMB* rs156429 with PD symptoms. One study revealed that rs156429 was associated with motor traits in older populations with mild parkinsonian signs (Shulman et al., 2014). Another result suggested that this SNP had no association with anxiety and depression symptoms in PD patients (Xu et al., 2016). As for gender factor, previous literature reported that *GPNMB* rs156429 might have a protective role in male Chinese PD patients (Liu et al., 2015). In our study, we did not find genotype distribution differences of *GPNMB* rs156429 between male and female PD patients. However, analysis by genders showed that this SNP might have a trend for being associated with cognitive dysfunction and pain symptoms in female PD populations. These clinical results could serve as clinical evidence for further exploring potential molecular mechanisms of *GPNMB* rs156429 in PD, especially its roles in PD pathogenesis and symptoms associated with gender differences.

GPNMB, also known as osteoactivin, is a glycoprotein widely expressed in a mass of tissues with multiple physiological functions. In addition to functioning as a metastatic mediator (Zhou et al., 2012; Maric et al., 2013), GPNMB could also play an important role in mediating bone mineral deposition (Abdelmagid et al., 2008; Zhuo and Zhou, 2016), contributing to tissue damage and inflammation (Ahn et al., 2002; Ripoll et al., 2007), and functioning in neuronal survival and neuroprotection (Huang et al., 2012; Srinivasan et al., 2016; Neal et al., 2018). So far, *GPNMB* has been reported to be involved in cancer diseases (Onaga et al., 2003; Kuan et al., 2006; Rose et al., 2010), non-alcoholic steatohepatitis (Katayama et al., 2015), inflammatory bowel disease (IBD), Niemann Pick disease-type C (Marques et al., 2016), Gaucher disease (Murugesan et al., 2018), amyotrophic lateral sclerosis (ALS) (Tanaka et al., 2012; Nagahara et al., 2015) and PD. In normal rat brain, GPNMB was widely expressed in cerebral cortex, striatum, olfactory bulb, hippocampus and other brain areas (Huang et al., 2012). In PD cases, it was reported that the transcription and expression levels of GPNMB were both increased in substantia nigra (Murthy et al., 2017; Moloney et al., 2018; Neal et al., 2018). Additionally, GPNMB was shown to interact with CD44, and CD44 expression was also highly increased after lipopolysaccharide (LPS) administration in rat substantia nigra (Ailane et al., 2013) and 1-methyl-4-phenyl-1,2,3,6-tetrahydropyridine (MPTP) treatment in mice (Kurkowska-Jastrzebska et al., 1999). A recent paper also found that overexpression of GPNMB could protect against neurodegeneration changes induced by MPTP (Budge et al., 2020). These evidences indicated that GPNMB could serve as an emerging target for neurodegenerative diseases (Budge et al., 2018).

Nearly 50% of PD patients were affected by cognitive dysfunction symptoms within 10 years of diagnosis (Williams-Gray et al., 2013). However, both the severity and timing vary among PD patients. Therefore, it will be of great clinical benefits for physicians to early identify patients at risk or at early stages of cognitive dysfunction. The precise mechanisms of cognitive impairment in PD still remain unknown, but it was widely acknowledged that the combination of pathological protein accumulations (alpha synuclein, tau, and amyloid beta) and neurotransmitter changes plays an important role in cognitive involvement (Weil et al., 2018). The association between GPNMB and PD cognitive function has not been reported, but its function in Alzheimer's disease (AD), has been profoundly studied. Firstly, GPNMB was found to be elevated in both cerebrospinal fluid (CSF) and brain samples of sporadic AD patients compared with cognitively healthy controls (Huttenrauch et al., 2018). This study further found that GPNMB was partially involved in microglia activation. Another study revealed that overexpressing GPNMB in transgenic mice could improve some hippocampal memory tests, along with increased levels of α-amino-3-hydroxy-5-methylisoxazole-4-propionate (AMPA) receptor subunit GluA1 in the hippocampus (Murata et al., 2015). In our study, we discovered that *GPNMB* rs156429 might have a trend for being associated with cognitive dysfunction in female PD patients. Therefore, the exact function of GPNMB in cognitive fields, and determination whether it functions differently between male and female, deserve further detailed investigations.

Pain, as a common non-motor symptom of PD, could greatly affect patients' quality of life. Studies proved that pain thresholds of PD tended to decrease, but this cause was not sufficient enough to explain pain development (Zambito Marsala et al., 2011). The mechanisms of pain symptoms in PD are quite complex, involving changes of cerebral brain, spinal cord, and peripheral nociceptive compartments (Rukavina et al., 2019). Several SNP variations have been identified to be involved in pain perception in PD patients like rs4680 and rs6267 of catechol-O-methyltransferase gene (*COMT*) (Lin et al., 2017), rs6746030 of sodium voltage-gated channel alpha subunit 9 gene (*SCN9A*), rs324419 of fatty acid amide hydrolase gene (*FAAH*) (Greenbaum et al., 2012), and *PINK1* (Gierthmuhlen et al., 2009). But *GPNMB* rs156429 has not yet been reported. Evidence suggests that the loss of dopaminergic neurons could lead to nociceptive hypersensitivity. One previous study reported that GPNMB inhibition could attenuate nociception in neuropathic pain mouse model (Hou et al., 2015). Our results indicated that *GPNMB* rs156429 might have a trend for being associated with pain symptoms in female PD patients. Therefore, our findings provided clinical evidence that *GPNMB* rs156429 might be involved in PD pain symptoms, especially in female patients, suggesting a possible biological implication of *GPNMB* rs156429 in PD manifestation development.

There were quite few literatures exploring possible mechanisms of GPNMB and gender differences. One study suggested that *GPNMB* could be an androgen-dysregulated gene (Tsui et al., 2012), and another indicated that estrogen was necessary but not sufficient for the expression of GPNMB (Prizant et al., 2016). In Liu's study, it was found that *GPNMB* rs156429 might have a protective role in male Chinese PD patients referring to PD onset when compared with healthy controls (Liu et al., 2015). They reported that male PD patients had significant lower frequency of G allele. In our study, we focused on exploring the possible effects of *GPNMB* rs156429 on PD symptoms. Therefore, healthy participants were not included. We applied covariance analysis to analyze the associations between scores of MMSE/MoCA and *GPNMB* rs156429 alleles in PD patients based on genders. We found that gender, rs156429 genotypes, and the interactions of gender and rs156429 genotypes all had statistically significant effects on MMSE and MoCA (*p* < 0.001). We then used Mantel-Haenszel statistics to investigate the association between pain symptoms and rs156429 alleles in PD patients based on genders. We found that T allele might be a risk factor for pain symptoms in female PD patients (OR = 1.14, 95% CI = 1.02–1.29). Our study and Liu's report focused on different aspects of *GPNMB* rs156429 and PD. G allele might be protective to male PD patients referring to PD onset in Liu's report, while T allele was likely to be a risk factor for pain symptoms in female PD patients in our study. These were not contradictory. According to these two studies, *GPNMB* rs156429 might have gender-based association or trend of association with PD onset or some PD symptoms, which could enable us to investigate why and how *GPNMB* rs156429 functions differently in PD based on genders.

There were some limitations in our study. Firstly, as a single centered study, the sample size of this study (*N* = 511) was not large. In female PD patients, we found that *p*-values after Bonferroni correction failed to have statistical significances (*p* = 0.064 after correction, MMSE; *p* = 0.064 after correction, MoCA; pain symptoms, *p* = 0.063 after correction, overdominant model after adjustment). Subsequent studies with larger sample size are urgently needed. Secondly, we did not perform more objective tests to assess PD and its clinical symptoms, such as polysomnography (PSG) to diagnose RBD, etc. Thirdly, other loci of *GPNMB* were not included in this study. Fourthly, other confounding factors, such as course of disease, etc., were not taken into account in this study. Those limitations are recommended to be considered in futures studies.

In conclusion, our study for the first time found that *GPNMB* rs156429 might have a trend for being associated with cognitive dysfunction and pain symptoms in the southeastern female Chinese PD population. Further studies of a larger sample size are needed to confirm these findings.

## Data Availability Statement

The raw data supporting the conclusions of this article will be made available by the authors, without undue reservation.

## Ethics Statement

The studies involving human participants were reviewed and approved by the ethics committee of Ruijin Hospital affiliated to Shanghai Jiao Tong University School of Medicine. The patients/participants provided their written informed consent to participate in this study.

## Author Contributions

PZ, XS, and WZ collected and sorted the data of PD patients. YH and GH completed the genetic analysis. GL and JL performed the statistical analysis and drafted the manuscript. SCh, SCu, and YT designed and supervised this study, double-checked the statistical analysis, and revised the manuscript. All authors contributed to the article and approved the submitted version.

## Conflict of Interest

The authors declare that the research was conducted in the absence of any commercial or financial relationships that could be construed as a potential conflict of interest.

## Abbreviations

AD: Alzheimer's disease
ADL: activity of daily living
ALS: Amyotrophic lateral sclerosis
AMPA: α-amino-3-hydroxy-5-methylisoxazole-4-propionate
BPI: Brief Pain Inventory
CI: Confidence interval
COMT: catechol-O-methyltransferase
CSF: cerebrospinal fluid
EOPD: early-onset Parkinson's disease
ESS: Epworth Sleeping Scale
FAAH: fatty acid amide hydrolase
FSS: Fatigue Severity Scale
GPNMB: glycoprotein non-metastatic melanoma protein B
GWAS: genome-wide association studies
HAMA: Hamilton Anxiety Rating Scale
HAMD: Hamilton Depression Rating Scale
HWE: Hardy-Weinberg equilibrium
H-Y staging: Hoehn-Yahr staging
IBD: Inflammatory bowel disease
LOPD: late-onset Parkinson's disease
LPS: lipopolysaccharide
MDS: Movement Disorder Society
MDS-UPDRS: the Movement Disorder Society Unified Parkinson's Disease Rating Scale
MMSE: Mini-Mental State Examination
MoCA: Montreal Cognitive Assessment
MPTP: 1-methyl-4-phenyl-1,2,3,6-tetrahydropyridine
NMSS: Non-Motor Symptoms Scale
OR: Odds ratio
PCR: polymerase chain reaction
PD: Parkinson's disease
PDQ-39: 39-item Parkinson's disease Questionnaire
PDSS: Parkinson's disease Sleep Scale
PSG: polysomnography
RBD: Rapid Eye Movement Sleep Behavior Disorder
RBD-HK: Rapid Eye Movement Sleep Behavior Disorder Questionnaire-Hong Kong Version
SCN9A: sodium voltage-gated channel alpha subunit 9
SCOPA-AUT: Scales for Outcomes in Parkinson's disease-Autonomic Questionnaire
SD: standard deviation
SE: standard error
SNP: single nucleotide polymorphism
SS-16: Sniffin& Sticks-16.
