# Supplementary material for: The Association Analysis of GPNMB rs156429 With Clinical Manifestations in Chinese Population With Parkinson's Disease
Source: Front Genet. 2020 Sep 2;11:952. doi: 10.3389/fgene.2020.00952 (PMC7492656; doi:10.3389/fgene.2020.00952)
Supplement: Supplementary file 1 [file Data_Sheet_1.docx]

**Supplementary Table 1 PD rating scales information in three genotypes of *GPNMB*** **rs156429 in PD patients**

|  | TT genotype  (n = 304) | CT genotype  (n = 176) | CC genotype  (n = 31) |
| --- | --- | --- | --- |
| SS-16 (mean ± SD) | 5.70 (4.05) | 5.23 (4.28) | 4.59 (4.29) |
| HAMA (mean ± SD) | 5.84 (6.10) | 5.78 (5.54) | 4.87 (5.44) |
| HAMD (mean ± SD) | 5.02 (5.18) | 5.12 (5.13) | 4.45 (5.07) |
| BPI (mean ± SD) | 7.64 (11.32) | 9.50 (12.71) | 12.16 (15.65) |
| RBD-HK (mean ± SD) | 12.07 (16.28) | 12.50 (17.61) | 13.19 (15.85) |
| PDSS (mean ± SD) | 119.23 (23.08) | 117.52 (22.75) | 118.55 (19.18) |
| PDQ39 (mean ± SD) | 15.86 (17.58) | 13.14 (14.51) | 16.83 (21.17) |
| FSS (mean ± SD) | 22.98 (19.06) | 24.73 (20.77) | 25.00 (20.36) |
| ESS (mean ± SD) | 5.30 (5.79) | 5.47 (6.02) | 5.03 (7.10) |
| MDS-UPDRS (mean ± SD) | 43.49 (25.79) | 44.11 (25.77) | 47.64 (27.54) |
| PART I | 8.06 (6.10) | 8.10 (5.68) | 7.80 (5.32) |
| PART II | 10.17 (7.24) | 10.16 (6.76) | 11.24 (7.77) |
| PART III | 25.33 (16.66) | 26.42 (17.41) | 27.44 (17.79) |
| NMSS (mean ± SD) | 28.76 (33.93) | 25.51 (28.32) | 27.90 (31.88) |
| cardiovascular | 0.61 (2.04) | 0.45 (1.34) | 0.94 (3.29) |
| sleep | 6.26 (7.64) | 5.55 (7.10) | 4.77 (5.91) |
| mood disorder | 5.85 (11.26) | 4.94 (8.56) | 5.32 (8.56) |
| delusion | 0.53 (2.77) | 0.27 (1.09) | 0.16 (0.58) |
| attention | 2.16 (3.24) | 2.20 (3.43) | 2.35 (4.04) |
| gastrointestinal | 3.07 (5.16) | 2.38 (4.50) | 3.23 (4.65) |
| urinary | 4.71 (8.59) | 4.00 (6.98) | 3.81 (7.47) |
| sexual dysfunction | 1.05 (4.16) | 1.22 (4.02) | 1.35 (4.77) |
| others | 4.54 (6.25) | 4.29 (5.90) | 4.16 (6.22) |
| SCOPA-AUT (mean ± SD) | 8.30 (8.61) | 8.41 (9.09) | 8.26 (8.84) |
| gastrointestinal | 2.79 (3.43) | 2.72 (3.60) | 2.61 (3.33) |
| urinary | 3.10 (4.35) | 3.14 (4.19) | 2.90 (3.87) |
| cardiovascular | 0.33 (0.92) | 0.37 (1.02) | 0.32 (0.83) |
| skin | 1.54 (2.46) | 1.67 (2.59) | 1.03 (1.68) |
| sexual dysfunction | 0.32 (1.23) | 0.27 (1.10) | 0.19 (0.79) |
| MMSE (mean ± SD) | 27.18 (3.06) | 27.10 (3.08) | 25.50 (3.53) |
| MoCA (mean ± SD) | 23.39 (4.48) | 22.85 (4.75) | 20.15 (5.66) |
| BPI, brief pain inventory; ESS, Epworth Sleepiness Scale; FSS, Fatigue severity scale; HAMA, Hamilton anxiety rating scale; HAMD, Hamilton depression rating scale; MDS, movement disorders society; MMSE, Mini-Mental State Examination; MoCA, Montreal Cognitive Assessment; NMSS, Non-Motor Symptoms Scale; PDQ-39, 39-item Parkinson's Disease Questionnaire; PDSS, Parkinson's disease sleep scale; RBD-HK, rapid eye movement sleep behavior disorder questionnaire-Hong Kong version; SCOPA-AUT, Scales for Outcomes in Parkinson's Disease-Autonomic questionnaire; SD, standard deviation; SS-16, Sniffin' Sticks 16; UPDRS, Unified Parkinson's Disease Rating Scale | | | |

**Supplementary Table 2** **The association between PD symptoms and genetic models of *GPNMB* rs156429 in PD patients**

|  | Additive model | | | | | | Additive model (adjusted) ^a^ | | | | | |
| --- | --- | --- | --- | --- | --- | --- | --- | --- | --- | --- | --- | --- |
|  | beta | SE | *p* value | OR | 95% CI | *p* value ^b^ | beta | SE | *p* value | OR | 95% CI | *p* value ^b^ |
| dysphagia | 0.133 | 0.207 | 0.523 | 1.14 | (0.75, 1.70) | 1.000 | 0.170 | 0.221 | 0.442 | 1.19 | (0.76, 1.81) | 1.000 |
| sialorrhea | -0.005 | 0.151 | 0.971 | 0.99 | (0.74, 1.34) | 1.000 | 0.012 | 0.160 | 0.943 | 1.01 | (0.74, 1.38) | 1.000 |
| symptom “full very quickly” | 0.048 | 0.242 | 0.842 | 1.05 | (0.64, 1.66) | 1.000 | -0.050 | 0.249 | 0.842 | 0.95 | (0.57, 1.53) | 1.000 |
| constipation | -0.053 | 0.152 | 0.726 | 0.95 | (0.70, 1.27) | 1.000 | -0.073 | 0.161 | 0.650 | 0.93 | (0.68, 1.27) | 1.000 |
| nocturia | -0.047 | 0.146 | 0.748 | 0.95 | (0.72, 1.27) | 1.000 | -0.029 | 0.155 | 0.850 | 0.97 | (0.72, 1.32) | 1.000 |
| postural hypotension | 0.046 | 0.189 | 0.808 | 1.05 | (0.72, 1.51) | 1.000 | 0.022 | 0.193 | 0.909 | 1.02 | (0.69, 1.48) | 1.000 |
| daytime sweatiness | -0.061 | 0.168 | 0.714 | 0.94 | (0.67, 1.30) | 1.000 | -0.045 | 0.172 | 0.792 | 0.96 | (0.68, 1.33) | 1.000 |
| nocturnal sweatiness | -0.120 | 0.189 | 0.526 | 0.89 | (0.61, 1.27) | 1.000 | -0.137 | 0.192 | 0.476 | 0.87 | (0.59, 1.26) | 1.000 |
| light sensitivity | -0.624 | 0.410 | 0.128 | 0.54 | (0.22, 1.12) | 1.000 | -0.653 | 0.410 | 0.111 | 0.52 | (0.21, 1.09) | 1.000 |
| susceptible to cold | -0.037 | 0.198 | 0.853 | 0.96 | (0.65, 1.41) | 1.000 | -0.047 | 0.200 | 0.813 | 0.95 | (0.64, 1.40) | 1.000 |
| susceptible to heat | 0.022 | 0.213 | 0.920 | 1.02 | (0.66, 1.54) | 1.000 | 0.026 | 0.218 | 0.904 | 1.03 | (0.66, 1.56) | 1.000 |
| sexual dysfunction | -0.093 | 0.287 | 0.746 | 0.91 | (0.50, 1.56) | 1.000 | -0.016 | 0.296 | 0.957 | 0.98 | (0.53, 1.72) | 1.000 |
| hallucination | -0.132 | 0.315 | 0.676 | 0.88 | (0.45, 1.58) | 1.000 | -0.162 | 0.333 | 0.628 | 0.85 | (0.43, 1.59) | 1.000 |
| apathy | 0.069 | 0.183 | 0.707 | 1.07 | (0.74, 1.53) | 1.000 | 0.094 | 0.187 | 0.614 | 1.10 | (0.76, 1.58) | 1.000 |
| pain | 0.253 | 0.147 | 0.086 | 1.29 | (0.96, 1.72) | 1.000 | 0.252 | 0.155 | 0.103 | 1.29 | (0.95, 1.75) | 1.000 |
| urination disorders  (not nocturia) | -0.023 | 0.153 | 0.879 | 0.98 | (0.72, 1.32) | 1.000 | -0.028 | 0.161 | 0.860 | 0.97 | (0.71, 1.33) | 1.000 |
| fatigue | 0.149 | 0.146 | 0.309 | 1.16 | (0.87, 1.55) | 1.000 | 0.165 | 0.155 | 0.289 | 1.18 | (0.87, 1.60) | 1.000 |
| freezing of gait | 0.236 | 0.183 | 0.197 | 1.27 | (0.88, 1.81) | 1.000 | 0.245 | 0.199 | 0.219 | 1.28 | (0.86, 1.88) | 1.000 |
| tremor | -0.199 | 0.146 | 0.173 | 0.82 | (0.61, 1.09) | 1.000 | -0.149 | 0.156 | 0.338 | 0.86 | (0.63, 1.17) | 1.000 |
| hyposmia | 0.104 | 0.171 | 0.543 | 1.11 | (0.80, 1.56) | 1.000 | 0.085 | 0.174 | 0.625 | 1.09 | (0.78, 1.54) | 1.000 |
| probable RBD | 0.131 | 0.159 | 0.410 | 1.14 | (0.83, 1.56) | 1.000 | 0.135 | 0.165 | 0.413 | 1.14 | (0.83, 1.58) | 1.000 |
|  | Dominant model | | | | | | Dominant model (adjusted) ^a^ | | | | | |
|  | beta | SE | *p* value | OR | 95% CI | *p* value ^b^ | beta | SE | *p* value | OR | 95% CI | *p* value ^b^ |
| dysphagia | 0.153 | 0.262 | 0.560 | 1.16 | (0.69, 1.94) | 1.000 | 0.217 | 0.280 | 0.439 | 1.24 | (0.71, 2.15) | 1.000 |
| sialorrhea | 0.003 | 0.188 | 0.987 | 1.00 | (0.69, 1.45) | 1.000 | 0.051 | 0.199 | 0.798 | 1.05 | (0.71, 1.55) | 1.000 |
| symptom “full very quickly” | 0.079 | 0.302 | 0.793 | 1.08 | (0.59, 1.95) | 1.000 | 0.003 | 0.311 | 0.991 | 1.00 | (0.54, 1.84) | 1.000 |
| constipation | -0.146 | 0.189 | 0.440 | 0.86 | (0.60, 1.25) | 1.000 | -0.159 | 0.200 | 0.425 | 0.85 | (0.58, 1.26) | 1.000 |
| nocturia | -0.048 | 0.181 | 0.791 | 0.95 | (0.67, 1.36) | 1.000 | -0.020 | 0.193 | 0.918 | 0.98 | (0.67, 1.43) | 1.000 |
| postural hypotension | 0.098 | 0.236 | 0.678 | 1.10 | (0.69, 1.75) | 1.000 | 0.090 | 0.241 | 0.710 | 1.09 | (0.68, 1.75) | 1.000 |
| daytime sweatiness | 0.036 | 0.206 | 0.863 | 1.04 | (0.69, 1.55) | 1.000 | 0.062 | 0.212 | 0.772 | 1.06 | (0.70, 1.61) | 1.000 |
| nocturnal sweatiness | -0.239 | 0.233 | 0.306 | 0.79 | (0.49, 1.24) | 1.000 | -0.250 | 0.238 | 0.293 | 0.78 | (0.49, 1.23) | 1.000 |
| light sensitivity | -0.793 | 0.477 | 0.096 | 0.45 | (0.16, 1.09) | 1.000 | -0.824 | 0.479 | 0.086 | 0.44 | (0.16, 1.06) | 1.000 |
| susceptible to cold | 0.010 | 0.243 | 0.967 | 1.01 | (0.62, 1.62) | 1.000 | 0.013 | 0.247 | 0.959 | 1.01 | (0.62, 1.64) | 1.000 |
| susceptible to heat | 0.182 | 0.263 | 0.488 | 1.20 | (0.71, 2.01) | 1.000 | 0.218 | 0.270 | 0.420 | 1.24 | (0.73, 2.11) | 1.000 |
| sexual dysfunction | -0.110 | 0.352 | 0.754 | 0.90 | (0.44, 1.76) | 1.000 | -0.031 | 0.360 | 0.931 | 0.97 | (0.47, 1.95) | 1.000 |
| hallucination | -0.217 | 0.387 | 0.575 | 0.81 | (0.37, 1.69) | 1.000 | -0.201 | 0.414 | 0.628 | 0.82 | (0.35, 1.81) | 1.000 |
| apathy | 0.102 | 0.229 | 0.657 | 1.11 | (0.70, 1.73) | 1.000 | 0.152 | 0.235 | 0.518 | 1.16 | (0.73, 1.84) | 1.000 |
| pain | 0.417 | 0.183 | **0.023** | **1.52** | **(1.06, 2.18)** | 0.483 | 0.444 | 0.193 | **0.021** | **1.56** | **(1.07, 2.28)** | 0.441 |
| urination disorders  (not nocturia) | 0.032 | 0.189 | 0.864 | 1.03 | (0.71, 1.50) | 1.000 | 0.043 | 0.199 | 0.828 | 1.04 | (0.71, 1.54) | 1.000 |
| fatigue | 0.193 | 0.181 | 0.288 | 1.21 | (0.85, 1.73) | 1.000 | 0.227 | 0.192 | 0.239 | 1.25 | (0.86, 1.83) | 1.000 |
| freezing of gait | 0.239 | 0.233 | 0.306 | 1.27 | (0.80, 2.00) | 1.000 | 0.274 | 0.252 | 0.277 | 1.32 | (0.80, 2.16) | 1.000 |
| tremor | -0.205 | 0.181 | 0.259 | 0.82 | (0.57, 1.16) | 1.000 | -0.135 | 0.193 | 0.486 | 0.87 | (0.60, 1.28) | 1.000 |
| hyposmia | 0.130 | 0.208 | 0.534 | 1.14 | (0.76, 1.72) | 1.000 | 0.098 | 0.213 | 0.647 | 1.10 | (0.73, 1.68) | 1.000 |
| probable RBD | 0.116 | 0.200 | 0.562 | 1.12 | (0.76, 1.66) | 1.000 | 0.145 | 0.207 | 0.485 | 1.16 | (0.77, 1.73) | 1.000 |
|  | Recessive model | | | | | | Recessive model (adjusted) ^a^ | | | | | |
|  | beta | SE | *p* value | OR | 95% CI | *p* value ^b^ | beta | SE | *p* value | OR | 95% CI | *p* value ^b^ |
| dysphagia | 0.218 | 0.506 | 0.666 | 1.24 | (0.41, 3.11) | 1.000 | 0.202 | 0.542 | 0.709 | 1.22 | (0.38, 3.28) | 1.000 |
| sialorrhea | -0.049 | 0.387 | 0.900 | 0.95 | (0.43, 2.00) | 1.000 | -0.140 | 0.412 | 0.733 | 0.87 | (0.38, 1.92) | 1.000 |
| symptom “full very quickly” | -0.018 | 0.627 | 0.978 | 0.98 | (0.23, 2.91) | 1.000 | -0.339 | 0.666 | 0.611 | 0.71 | (0.16, 2.28) | 1.000 |
| constipation | 0.260 | 0.376 | 0.490 | 1.30 | (0.61, 2.69) | 1.000 | 0.192 | 0.401 | 0.632 | 1.21 | (0.54, 2.65) | 1.000 |
| nocturia | -0.102 | 0.372 | 0.784 | 0.90 | (0.43, 1.87) | 1.000 | -0.106 | 0.393 | 0.787 | 0.90 | (0.41, 1.95) | 1.000 |
| postural hypotension | -0.118 | 0.503 | 0.815 | 0.89 | (0.29, 2.20) | 1.000 | -0.237 | 0.517 | 0.647 | 0.79 | (0.25, 2.01) | 1.000 |
| daytime sweatiness | -0.632 | 0.499 | 0.205 | 0.53 | (0.18, 1.30) | 1.000 | -0.626 | 0.505 | 0.215 | 0.53 | (0.18, 1.33) | 1.000 |
| nocturnal sweatiness | 0.215 | 0.445 | 0.628 | 1.24 | (0.48, 2.83) | 1.000 | 0.147 | 0.454 | 0.746 | 1.16 | (0.44, 2.69) | 1.000 |
| light sensitivity | -0.461 | 1.038 | 0.657 | 0.63 | (0.03, 3.15) | 1.000 | -0.559 | 1.044 | 0.592 | 0.57 | (0.03, 2.90) | 1.000 |
| susceptible to cold | -0.305 | 0.550 | 0.579 | 0.74 | (0.21, 1.95) | 1.000 | -0.385 | 0.556 | 0.489 | 0.68 | (0.20, 1.83) | 1.000 |
| susceptible to heat | -0.843 | 0.743 | 0.257 | 0.43 | (0.07, 1.48) | 1.000 | -0.968 | 0.759 | 0.202 | 0.38 | (0.06, 1.35) | 1.000 |
| sexual dysfunction | -0.136 | 0.752 | 0.857 | 0.87 | (0.14, 3.07) | 1.000 | 0.034 | 0.766 | 0.965 | 1.03 | (0.16, 3.78) | 1.000 |
| hallucination | 0.066 | 0.756 | 0.931 | 1.07 | (0.17, 3.79) | 1.000 | -0.211 | 0.841 | 0.802 | 0.81 | (0.11, 3.38) | 1.000 |
| apathy | 0.020 | 0.469 | 0.965 | 1.02 | (0.37, 2.41) | 1.000 | -0.016 | 0.479 | 0.973 | 0.98 | (0.35, 2.37) | 1.000 |
| pain | -0.113 | 0.380 | 0.766 | 0.89 | (0.41, 1.86) | 1.000 | -0.224 | 0.396 | 0.571 | 0.80 | (0.36, 1.72) | 1.000 |
| urination disorders  (not nocturia) | -0.300 | 0.407 | 0.462 | 0.74 | (0.32, 1.60) | 1.000 | -0.380 | 0.428 | 0.374 | 0.68 | (0.28, 1.54) | 1.000 |
| fatigue | 0.152 | 0.372 | 0.682 | 1.16 | (0.56, 2.45) | 1.000 | 0.113 | 0.393 | 0.773 | 1.12 | (0.52, 2.47) | 1.000 |
| freezing of gait | 0.504 | 0.428 | 0.239 | 1.66 | (0.67, 3.69) | 1.000 | 0.428 | 0.478 | 0.370 | 1.53 | (0.57, 3.76) | 1.000 |
| tremor | -0.429 | 0.372 | 0.250 | 0.65 | (0.31, 1.35) | 1.000 | -0.394 | 0.392 | 0.315 | 0.67 | (0.31, 1.46) | 1.000 |
| hyposmia | 0.118 | 0.446 | 0.791 | 1.13 | (0.49, 2.91) | 1.000 | 0.137 | 0.455 | 0.763 | 1.15 | (0.49, 3.00) | 1.000 |
| probable RBD | 0.352 | 0.389 | 0.365 | 1.42 | (0.64, 3.00) | 1.000 | 0.264 | 0.407 | 0.516 | 1.30 | (0.57, 2.85) | 1.000 |
|  | Overdominant model | | | | | | Overdominant model (adjusted) ^a^ | | | | | |
|  | beta | SE | *p* value | OR | 95% CI | *p* value ^b^ | beta | SE | *p* value | OR | 95% CI | *p* value ^b^ |
| dysphagia | -0.104 | 0.270 | 0.700 | 0.90 | (0.53, 1.55) | 1.000 | -0.175 | 0.288 | 0.542 | 0.84 | (0.48, 1.49) | 1.000 |
| sialorrhea | -0.016 | 0.194 | 0.936 | 0.98 | (0.67, 1.44) | 1.000 | -0.089 | 0.205 | 0.663 | 0.91 | (0.61, 1.37) | 1.000 |
| symptom “full very quickly” | -0.089 | 0.311 | 0.776 | 0.92 | (0.50, 1.71) | 1.000 | -0.090 | 0.319 | 0.777 | 0.91 | (0.49, 1.74) | 1.000 |
| constipation | 0.226 | 0.197 | 0.252 | 1.25 | (0.85, 1.85) | 1.000 | 0.220 | 0.207 | 0.287 | 1.25 | (0.83, 1.88) | 1.000 |
| nocturia | 0.025 | 0.187 | 0.892 | 1.03 | (0.71, 1.48) | 1.000 | -0.006 | 0.199 | 0.975 | 0.99 | (0.67, 1.47) | 1.000 |
| postural hypotension | -0.132 | 0.242 | 0.584 | 0.88 | (0.55, 1.42) | 1.000 | -0.153 | 0.247 | 0.535 | 0.86 | (0.53, 1.40) | 1.000 |
| daytime sweatiness | -0.174 | 0.211 | 0.409 | 0.84 | (0.56, 1.27) | 1.000 | -0.203 | 0.217 | 0.349 | 0.82 | (0.53, 1.25) | 1.000 |
| nocturnal sweatiness | 0.320 | 0.246 | 0.193 | 1.38 | (0.86, 2.26) | 1.000 | 0.313 | 0.250 | 0.211 | 1.37 | (0.85, 2.26) | 1.000 |
| light sensitivity | 0.764 | 0.509 | 0.134 | 2.15 | (0.85, 6.54) | 1.000 | 0.769 | 0.510 | 0.132 | 2.16 | (0.85, 6.59) | 1.000 |
| susceptible to cold | -0.081 | 0.250 | 0.746 | 0.92 | (0.57, 1.52) | 1.000 | -0.104 | 0.253 | 0.681 | 0.90 | (0.55, 1.49) | 1.000 |
| susceptible to heat | -0.347 | 0.266 | 0.193 | 0.71 | (0.42, 1.20) | 1.000 | -0.412 | 0.273 | 0.132 | 0.66 | (0.39, 1.14) | 1.000 |
| sexual dysfunction | 0.085 | 0.364 | 0.816 | 1.09 | (0.54, 2.30) | 1.000 | 0.041 | 0.373 | 0.912 | 1.04 | (0.51, 2.23) | 1.000 |
| hallucination | 0.254 | 0.407 | 0.533 | 1.29 | (0.60, 3.01) | 1.000 | 0.160 | 0.432 | 0.712 | 1.17 | (0.52, 2.86) | 1.000 |
| apathy | -0.103 | 0.236 | 0.661 | 0.90 | (0.57, 1.44) | 1.000 | -0.165 | 0.241 | 0.493 | 0.85 | (0.53, 1.37) | 1.000 |
| pain | -0.473 | 0.189 | **0.012** | **0.62** | **(0.43, 0.90)** | 0.252 | -0.531 | 0.199 | **0.008** | **0.59** | **(0.40, 0.87)** | 0.168 |
| urination disorders  (not nocturia) | -0.107 | 0.195 | 0.583 | 0.90 | (0.61, 1.32) | 1.000 | -0.139 | 0.205 | 0.499 | 0.87 | (0.58, 1.30) | 1.000 |
| fatigue | -0.167 | 0.187 | 0.372 | 0.85 | (0.59, 1.22) | 1.000 | -0.213 | 0.199 | 0.284 | 0.81 | (0.55, 1.19) | 1.000 |
| freezing of gait | -0.111 | 0.241 | 0.645 | 0.89 | (0.56, 1.45) | 1.000 | -0.173 | 0.259 | 0.505 | 0.84 | (0.51, 1.41) | 1.000 |
| tremor | 0.109 | 0.187 | 0.558 | 1.12 | (0.77, 1.61) | 1.000 | 0.042 | 0.200 | 0.835 | 1.04 | (0.70, 1.54) | 1.000 |
| hyposmia | -0.110 | 0.215 | 0.609 | 0.90 | (0.58, 1.36) | 1.000 | -0.071 | 0.220 | 0.746 | 0.93 | (0.60, 1.43) | 1.000 |
| probable RBD | -0.029 | 0.207 | 0.888 | 0.97 | (0.65, 1.46) | 1.000 | -0.083 | 0.214 | 0.698 | 0.92 | (0.61, 1.41) | 1.000 |
| CI: confidence interval; OR: odds ratio; RBD: Rapid eye movement sleep behavior disorder; SE, standard error  a Hoehn-Yahr staging and gender were taken as adjustments  b *p* value after Bonferroni correction  Bond fonts: *p* < 0.05 | | | | | | | | | | | | |

**Supplementary Table 3** **The association between PD rating scales and genotype of *GPNMB* rs156429 in female PD patients**

|  | *p* value | *p* value ^a^ | *p* value  (multiple pairwise-comparison between groups) | | |
| --- | --- | --- | --- | --- | --- |
|  | TT v.s. CT v.s. CC  (n = 133 v.s. 80 v.s. 18) | TT v.s. CT v.s. CC  (n = 133 v.s. 80 v.s. 18) | TT v.s. CT  (n = 133 v.s. 80) | TT v.s. CC  (n = 133 v.s. 18) | CT v.s. CC  (n = 80 v.s. 18) |
| SS-16 | 0.278 | 1.000 | 0.467 | 0.333 | 0.467 |
| HAMA | 0.887 | 1.000 | 0.921 | 0.921 | 0.921 |
| HAMD | 0.830 | 1.000 | 0.914 | 0.863 | 0.863 |
| BPI | 0.197 | 1.000 | 0.310 | 0.310 | 0.767 |
| RBD-HK | 0.370 | 1.000 | 0.873 | 0.285 | 0.285 |
| PDSS | 0.258 | 1.000 | 0.430 | 0.430 | 0.967 |
| PDQ39 | 0.516 | 1.000 | 0.500 | 0.500 | 0.500 |
| FSS | 0.680 | 1.000 | 0.738 | 0.738 | 0.738 |
| ESS | 0.678 | 1.000 | 0.796 | 0.642 | 0.642 |
| MDS-UPDRS | 0.345 | 1.000 | 0.563 | 0.567 | 0.900 |
| PART I | 0.421 | 1.000 | 0.611 | 0.821 | 0.962 |
| PART II | 0.053 | 1.000 | 0.192 | 0.093 | 0.192 |
| PART III | 0.413 | 1.000 | 0.508 | 0.508 | 0.614 |
| NMSS | 0.794 | 1.000 | 0.954 | 0.832 | 0.832 |
| cardiovascular | 0.977 | 1.000 | 0.927 | 0.927 | 0.927 |
| sleep | 0.947 | 1.000 | 0.988 | 0.988 | 0.988 |
| mood disorder | 0.698 | 1.000 | 0.899 | 0.635 | 0.635 |
| delusion | 0.353 | 1.000 | 0.395 | 0.521 | 0.395 |
| attention | 0.903 | 1.000 | 0.841 | 0.841 | 0.841 |
| gastrointestinal | 0.249 | 1.000 | 0.579 | 0.254 | 0.254 |
| urinary | 0.540 | 1.000 | 0.583 | 0.583 | 0.760 |
| sexual dysfunction | 0.628 | 1.000 | 0.673 | 0.673 | 0.794 |
| others | 0.966 | 1.000 | 0.958 | 0.958 | 0.958 |
| SCOPA-AUT | 0.808 | 1.000 | 0.981 | 0.805 | 0.805 |
| gastrointestinal | 0.670 | 1.000 | 0.600 | 0.600 | 0.600 |
| urinary | 0.430 | 1.000 | 0.505 | 0.505 | 0.505 |
| cardiovascular | 0.938 | 1.000 | 0.876 | 0.876 | 0.876 |
| skin | 0.985 | 1.000 | 0.979 | 0.979 | 0.979 |
| sexual dysfunction | 0.695 | 1.000 | 0.755 | 0.693 | 0.693 |
| drug usage | 0.353 | 1.000 | 0.779 | 0.354 | 0.354 |
| MMSE | **0.002** | 0.064 | **0.045** | **0.004** | **0.024** |
| MoCA | **0.002** | 0.064 | **0.043** | **0.006** | 0.071 |
| BPI, brief pain inventory; ESS, Epworth Sleepiness Scale; FSS, Fatigue severity scale; HAMA, Hamilton anxiety rating scale; HAMD, Hamilton depression rating scale; MDS, movement disorders society; MMSE, Mini-Mental State Examination; MoCA, Montreal Cognitive Assessment; NMSS, Non-Motor Symptoms Scale; PDQ-39, 39-item Parkinson's Disease Questionnaire; PDSS, Parkinson's disease sleep scale; RBD-HK, rapid eye movement sleep behavior disorder questionnaire-Hong Kong version; SCOPA-AUT, Scales for Outcomes in Parkinson's Disease-Autonomic questionnaire; SS-16, Sniffin' Sticks 16; UPDRS, Unified Parkinson's Disease Rating Scale  a *p* value after Bonferroni correction  Bond fonts: *p* < 0.05 | | | | | |

**Supplementary Table 4 The association between PD symptoms and genetic models of *GPNMB* rs156429 in female PD patients**

|  | Additive model | | | | | | Additive model (adjusted) ^a^ | | | | | |
| --- | --- | --- | --- | --- | --- | --- | --- | --- | --- | --- | --- | --- |
|  | beta | SE | *p* value | OR | 95% CI | *p* value ^b^ | beta | SE | *p* value | OR | 95% CI | *p* value ^b^ |
| dysphagia | 0.267 | 0.315 | 0.398 | 1.31 | (0.69, 2.39) | 1.000 | 0.172 | 0.322 | 0.593 | 1.19 | (0.62, 2.20) | 1.000 |
| sialorrhea | 0.122 | 0.213 | 0.565 | 1.13 | (0.74, 1.71) | 1.000 | 0.027 | 0.228 | 0.904 | 1.03 | (0.65, 1.61) | 1.000 |
| symptom “full very quickly” | -0.090 | 0.313 | 0.774 | 0.91 | (0.48, 1.65) | 1.000 | -0.176 | 0.318 | 0.580 | 0.84 | (0.43, 1.53) | 1.000 |
| constipation | 0.260 | 0.213 | 0.222 | 1.30 | (0.85, 1.97) | 1.000 | 0.190 | 0.226 | 0.401 | 1.21 | (0.77, 1.89) | 1.000 |
| nocturia | 0.336 | 0.210 | 0.109 | 1.40 | (0.93, 2.12) | 1.000 | 0.335 | 0.219 | 0.127 | 1.40 | (0.91, 2.16) | 1.000 |
| postural hypotension | 0.110 | 0.260 | 0.673 | 1.12 | (0.66, 1.84) | 1.000 | 0.055 | 0.265 | 0.836 | 1.06 | (0.62, 1.76) | 1.000 |
| daytime sweatiness | -0.053 | 0.246 | 0.830 | 0.95 | (0.58, 1.52) | 1.000 | -0.059 | 0.250 | 0.814 | 0.94 | (0.57, 1.53) | 1.000 |
| nocturnal sweatiness | -0.093 | 0.261 | 0.720 | 0.91 | (0.54, 1.50) | 1.000 | -0.136 | 0.265 | 0.606 | 0.87 | (0.51, 1.45) | 1.000 |
| light sensitivity | -0.097 | 0.459 | 0.833 | 0.91 | (0.34, 2.11) | 1.000 | -0.100 | 0.463 | 0.830 | 0.91 | (0.33, 2.12) | 1.000 |
| susceptible to cold | -0.030 | 0.272 | 0.912 | 0.97 | (0.56, 1.63) | 1.000 | -0.075 | 0.275 | 0.786 | 0.93 | (0.53, 1.57) | 1.000 |
| susceptible to heat | 0.098 | 0.301 | 0.744 | 1.10 | (0.60, 1.96) | 1.000 | -0.004 | 0.309 | 0.991 | 1.00 | (0.53, 1.80) | 1.000 |
| sexual dysfunction | -0.185 | 0.635 | 0.771 | 0.83 | (0.19, 2.57) | 1.000 | -0.274 | 0.627 | 0.662 | 0.76 | (0.18, 2.34) | 1.000 |
| hallucination | -0.097 | 0.459 | 0.833 | 0.91 | (0.34, 2.11) | 1.000 | -0.216 | 0.459 | 0.638 | 0.81 | (0.30, 1.88) | 1.000 |
| apathy | 0.243 | 0.256 | 0.342 | 1.28 | (0.76, 2.09) | 1.000 | 0.204 | 0.260 | 0.431 | 1.23 | (0.73, 2.03) | 1.000 |
| pain | 0.357 | 0.210 | 0.089 | 1.43 | (0.95, 2.17) | 1.000 | 0.330 | 0.220 | 0.134 | 1.39 | (0.91, 2.16) | 1.000 |
| urination disorders  (not nocturia) | 0.080 | 0.219 | 0.714 | 1.08 | (0.70, 1.66) | 1.000 | -0.005 | 0.230 | 0.984 | 1.00 | (0.63, 1.56) | 1.000 |
| fatigue | 0.227 | 0.209 | 0.277 | 1.26 | (0.84, 1.90) | 1.000 | 0.209 | 0.222 | 0.348 | 1.23 | (0.80, 1.92) | 1.000 |
| freezing of gait | 0.533 | 0.259 | 0.039 | 1.70 | (1.02, 2.83) | 0.819 | 0.458 | 0.288 | 0.112 | 1.58 | (0.89, 2.79) | 1.000 |
| tremor | -0.012 | 0.209 | 0.952 | 0.99 | (0.66, 1.49) | 1.000 | 0.023 | 0.222 | 0.918 | 1.02 | (0.66, 1.59) | 1.000 |
| hyposmia | 0.205 | 0.243 | 0.399 | 1.23 | (0.77, 2.01) | 1.000 | 0.187 | 0.249 | 0.452 | 1.21 | (0.75, 1.99) | 1.000 |
| probable RBD | 0.284 | 0.219 | 0.194 | 1.33 | (0.86, 2.04) | 1.000 | 0.252 | 0.230 | 0.274 | 1.29 | (0.82, 2.02) | 1.000 |
|  | Dominant model | | | | | | Dominant model (adjusted) ^a^ | | | | | |
|  | beta | SE | *p* value | OR | 95% CI | *p* value ^b^ | beta | SE | *p* value | OR | 95% CI | *p* value ^b^ |
| dysphagia | 0.277 | 0.425 | 0.515 | 1.32 | (0.57, 3.05) | 1.000 | 0.214 | 0.434 | 0.622 | 1.24 | (0.52, 2.91) | 1.000 |
| sialorrhea | 0.105 | 0.277 | 0.705 | 1.11 | (0.64, 1.91) | 1.000 | 0.015 | 0.294 | 0.961 | 1.01 | (0.57, 1.80) | 1.000 |
| symptom “full very quickly” | -0.091 | 0.399 | 0.819 | 0.91 | (0.41, 1.98) | 1.000 | -0.160 | 0.407 | 0.694 | 0.85 | (0.38, 1.87) | 1.000 |
| constipation | 0.093 | 0.278 | 0.737 | 1.10 | (0.63, 1.89) | 1.000 | 0.010 | 0.293 | 0.973 | 1.01 | (0.57, 1.79) | 1.000 |
| nocturia | 0.328 | 0.270 | 0.225 | 1.39 | (0.82, 2.36) | 1.000 | 0.335 | 0.282 | 0.235 | 1.40 | (0.81, 2.43) | 1.000 |
| postural hypotension | 0.114 | 0.341 | 0.738 | 1.12 | (0.57, 2.18) | 1.000 | 0.066 | 0.347 | 0.849 | 1.07 | (0.54, 2.10) | 1.000 |
| daytime sweatiness | 0.036 | 0.315 | 0.909 | 1.04 | (0.56, 1.92) | 1.000 | 0.046 | 0.322 | 0.886 | 1.05 | (0.55, 1.96) | 1.000 |
| nocturnal sweatiness | -0.189 | 0.335 | 0.573 | 0.83 | (0.42, 1.59) | 1.000 | -0.233 | 0.340 | 0.493 | 0.79 | (0.40, 1.53) | 1.000 |
| light sensitivity | -0.153 | 0.586 | 0.795 | 0.86 | (0.25, 2.66) | 1.000 | -0.159 | 0.589 | 0.788 | 0.85 | (0.25, 2.66) | 1.000 |
| susceptible to cold | -0.146 | 0.353 | 0.678 | 0.86 | (0.43, 1.71) | 1.000 | -0.191 | 0.357 | 0.593 | 0.83 | (0.40, 1.65) | 1.000 |
| susceptible to heat | 0.222 | 0.393 | 0.573 | 1.25 | (0.57, 2.70) | 1.000 | 0.156 | 0.404 | 0.700 | 1.17 | (0.52, 2.58) | 1.000 |
| sexual dysfunction | 0.040 | 0.776 | 0.959 | 1.04 | (0.20, 4.83) | 1.000 | -0.024 | 0.781 | 0.976 | 0.98 | (0.19, 4.57) | 1.000 |
| hallucination | -0.153 | 0.586 | 0.795 | 0.86 | (0.25, 2.66) | 1.000 | -0.234 | 0.597 | 0.695 | 0.79 | (0.23, 2.50) | 1.000 |
| apathy | 0.346 | 0.340 | 0.309 | 1.41 | (0.72, 2.76) | 1.000 | 0.313 | 0.344 | 0.364 | 1.37 | (0.69, 2.69) | 1.000 |
| pain | 0.684 | 0.272 | **0.012** | **1.98** | **(1.17, 3.39)** | 0.252 | 0.687 | 0.285 | **0.016** | **1.99** | **(1.14, 3.49)** | 0.336 |
| urination disorders  (not nocturia) | 0.207 | 0.284 | 0.466 | 1.23 | (0.70, 2.15) | 1.000 | 0.143 | 0.297 | 0.630 | 1.15 | (0.64, 2.06) | 1.000 |
| fatigue | 0.254 | 0.269 | 0.344 | 1.29 | (0.76, 2.19) | 1.000 | 0.247 | 0.284 | 0.384 | 1.28 | (0.73, 2.24) | 1.000 |
| freezing of gait | 0.582 | 0.354 | 0.100 | 1.79 | (0.89, 3.61) | 1.000 | 0.549 | 0.386 | 0.155 | 1.73 | (0.81, 3.73) | 1.000 |
| tremor | 0.000 | 0.269 | 1.000 | 1.00 | (0.59, 1.70) | 1.000 | 0.029 | 0.286 | 0.919 | 1.03 | (0.59, 1.81) | 1.000 |
| hyposmia | 0.175 | 0.305 | 0.565 | 1.19 | (0.66, 2.19) | 1.000 | 0.151 | 0.312 | 0.629 | 1.16 | (0.63, 2.16) | 1.000 |
| probable RBD | 0.207 | 0.287 | 0.471 | 1.23 | (0.70, 2.16) | 1.000 | 0.192 | 0.300 | 0.523 | 1.21 | (0.67, 2.18) | 1.000 |
|  | Recessive model | | | | | | Recessive model (adjusted) ^a^ | | | | | |
|  | beta | SE | *p* value | OR | 95% CI | *p* value ^b^ | beta | SE | *p* value | OR | 95% CI | *p* value ^b^ |
| dysphagia | 0.541 | 0.671 | 0.420 | 1.72 | (0.38, 5.73) | 1.000 | 0.265 | 0.714 | 0.710 | 1.30 | (0.26, 4.69) | 1.000 |
| sialorrhea | 0.331 | 0.495 | 0.504 | 1.39 | (0.51, 3.68) | 1.000 | 0.105 | 0.539 | 0.846 | 1.11 | (0.37, 3.19) | 1.000 |
| symptom “full very quickly” | -0.202 | 0.777 | 0.795 | 0.82 | (0.12, 3.08) | 1.000 | -0.477 | 0.814 | 0.558 | 0.62 | (0.09, 2.51) | 1.000 |
| constipation | 1.110 | 0.505 | **0.028** | **3.03** | **(1.15, 8.56)** | 0.588 | 1.026 | 0.547 | 0.061 | 2.79 | (0.98, 8.66) | 1.000 |
| nocturia | 0.787 | 0.503 | 0.118 | 2.20 | (0.83, 6.18) | 1.000 | 0.757 | 0.532 | 0.155 | 2.13 | (0.77, 6.44) | 1.000 |
| postural hypotension | 0.231 | 0.594 | 0.697 | 1.26 | (0.34, 3.74) | 1.000 | 0.088 | 0.612 | 0.886 | 1.09 | (0.29, 3.36) | 1.000 |
| daytime sweatiness | -0.466 | 0.653 | 0.475 | 0.63 | (0.14, 2.00) | 1.000 | -0.534 | 0.663 | 0.421 | 0.59 | (0.13, 1.91) | 1.000 |
| nocturnal sweatiness | 0.110 | 0.592 | 0.853 | 1.12 | (0.30, 3.30) | 1.000 | 0.019 | 0.604 | 0.975 | 1.02 | (0.27, 3.09) | 1.000 |
| light sensitivity | -0.025 | 1.071 | 0.982 | 0.98 | (0.05, 5.43) | 1.000 | -0.016 | 1.078 | 0.988 | 0.98 | (0.05, 5.59) | 1.000 |
| susceptible to cold | 0.295 | 0.595 | 0.620 | 1.34 | (0.37, 4.00) | 1.000 | 0.200 | 0.608 | 0.742 | 1.22 | (0.32, 3.73) | 1.000 |
| susceptible to heat | -0.202 | 0.777 | 0.795 | 0.82 | (0.12, 3.08) | 1.000 | -0.584 | 0.836 | 0.484 | 0.56 | (0.08, 2.34) | 1.000 |
| sexual dysfunction | -- | -- | 0.992 | -- | -- | 1.000 | -- | -- | 0.992 | -- | -- | 1.000 |
| hallucination | -0.025 | 1.071 | 0.982 | 0.98 | (0.05, 5.43) | 1.000 | -0.463 | 1.144 | 0.686 | 0.63 | (0.03, 4.00) | 1.000 |
| apathy | 0.231 | 0.594 | 0.697 | 1.26 | (0.34, 3.74) | 1.000 | 0.128 | 0.608 | 0.834 | 1.14 | (0.30, 3.47) | 1.000 |
| pain | -0.290 | 0.503 | 0.563 | 0.75 | (0.27, 1.97) | 1.000 | -0.454 | 0.523 | 0.385 | 0.63 | (0.22, 1.75) | 1.000 |
| urination disorders  (not nocturia) | -0.255 | 0.546 | 0.640 | 0.77 | (0.24, 2.14) | 1.000 | -0.539 | 0.586 | 0.357 | 0.58 | (0.17, 1.74) | 1.000 |
| fatigue | 0.424 | 0.503 | 0.400 | 1.53 | (0.58, 4.29) | 1.000 | 0.335 | 0.534 | 0.531 | 1.40 | (0.50, 4.24) | 1.000 |
| freezing of gait | 0.992 | 0.535 | 0.064 | 2.70 | (0.89, 7.48) | 1.000 | 0.720 | 0.624 | 0.248 | 2.06 | (0.56, 6.72) | 1.000 |
| tremor | -0.070 | 0.494 | 0.887 | 0.93 | (0.35, 2.53) | 1.000 | 0.030 | 0.525 | 0.955 | 1.03 | (0.37, 3.02) | 1.000 |
| hyposmia | 0.614 | 0.655 | 0.348 | 1.85 | (0.58, 8.22) | 1.000 | 0.591 | 0.663 | 0.373 | 1.81 | (0.55, 8.13) | 1.000 |
| probable RBD | 0.854 | 0.495 | 0.084 | 2.35 | (0.88, 6.29) | 1.000 | 0.739 | 0.529 | 0.162 | 2.09 | (0.73, 6.01) | 1.000 |
|  | Overdominant model | | | | | | Overdominant model (adjusted) ^a^ | | | | | |
|  | beta | SE | *p* value | OR | 95% CI | *p* value ^b^ | beta | SE | *p* value | OR | 95% CI | *p* value ^b^ |
| dysphagia | -0.096 | 0.442 | 0.828 | 0.91 | (0.39, 2.25) | 1.000 | -0.132 | 0.452 | 0.770 | 0.88 | (0.37, 2.20) | 1.000 |
| sialorrhea | -0.004 | 0.289 | 0.989 | 1.00 | (0.57, 1.76) | 1.000 | 0.018 | 0.304 | 0.954 | 1.02 | (0.56, 1.86) | 1.000 |
| symptom “full very quickly” | 0.037 | 0.415 | 0.928 | 1.04 | (0.47, 2.43) | 1.000 | 0.026 | 0.422 | 0.951 | 1.03 | (0.46, 2.43) | 1.000 |
| constipation | 0.279 | 0.295 | 0.343 | 1.32 | (0.75, 2.38) | 1.000 | 0.324 | 0.308 | 0.293 | 1.38 | (0.76, 2.55) | 1.000 |
| nocturia | -0.101 | 0.281 | 0.719 | 0.90 | (0.52, 1.57) | 1.000 | -0.120 | 0.292 | 0.682 | 0.89 | (0.50, 1.57) | 1.000 |
| postural hypotension | -0.045 | 0.356 | 0.900 | 0.96 | (0.48, 1.96) | 1.000 | -0.041 | 0.361 | 0.909 | 0.96 | (0.48, 1.98) | 1.000 |
| daytime sweatiness | -0.171 | 0.324 | 0.598 | 0.84 | (0.45, 1.61) | 1.000 | -0.203 | 0.330 | 0.539 | 0.82 | (0.43, 1.58) | 1.000 |
| nocturnal sweatiness | 0.246 | 0.355 | 0.489 | 1.28 | (0.65, 2.63) | 1.000 | 0.262 | 0.359 | 0.465 | 1.30 | (0.65, 2.69) | 1.000 |
| light sensitivity | 0.159 | 0.618 | 0.797 | 1.17 | (0.37, 4.44) | 1.000 | 0.168 | 0.620 | 0.786 | 1.18 | (0.37, 4.50) | 1.000 |
| susceptible to cold | 0.268 | 0.376 | 0.476 | 1.31 | (0.64, 2.82) | 1.000 | 0.283 | 0.380 | 0.456 | 1.33 | (0.64, 2.88) | 1.000 |
| susceptible to heat | -0.295 | 0.402 | 0.463 | 0.74 | (0.34, 1.67) | 1.000 | -0.338 | 0.413 | 0.412 | 0.71 | (0.32, 1.63) | 1.000 |
| sexual dysfunction | -0.385 | 0.777 | 0.620 | 0.68 | (0.15, 3.53) | 1.000 | -0.436 | 0.788 | 0.581 | 0.65 | (0.13, 3.40) | 1.000 |
| hallucination | 0.159 | 0.618 | 0.797 | 1.17 | (0.37, 4.44) | 1.000 | 0.101 | 0.628 | 0.872 | 1.11 | (0.34, 4.26) | 1.000 |
| apathy | -0.293 | 0.349 | 0.402 | 0.75 | (0.38, 1.50) | 1.000 | -0.291 | 0.353 | 0.410 | 0.75 | (0.38, 1.51) | 1.000 |
| pain | -0.835 | 0.285 | **0.003** | **0.43** | **(0.25, 0.75)** | 0.063 | -0.898 | 0.301 | **0.003** | **0.41** | **(0.22, 0.73)** | 0.063 |
| urination disorders  (not nocturia) | -0.301 | 0.294 | 0.305 | 0.74 | (0.42, 1.32) | 1.000 | -0.313 | 0.306 | 0.306 | 0.73 | (0.40, 1.34) | 1.000 |
| fatigue | -0.141 | 0.279 | 0.614 | 0.87 | (0.50, 1.50) | 1.000 | -0.162 | 0.295 | 0.584 | 0.85 | (0.47, 1.51) | 1.000 |
| freezing of gait | -0.231 | 0.363 | 0.525 | 0.79 | (0.39, 1.65) | 1.000 | -0.318 | 0.392 | 0.418 | 0.73 | (0.34, 1.59) | 1.000 |
| tremor | -0.022 | 0.280 | 0.936 | 0.98 | (0.56, 1.69) | 1.000 | -0.022 | 0.296 | 0.942 | 0.98 | (0.54, 1.75) | 1.000 |
| hyposmia | -0.022 | 0.315 | 0.945 | 0.98 | (0.52, 1.80) | 1.000 | -0.001 | 0.322 | 0.999 | 1.00 | (0.53, 1.87) | 1.000 |
| probable RBD | 0.076 | 0.301 | 0.802 | 1.08 | (0.60, 1.96) | 1.000 | 0.044 | 0.313 | 0.888 | 1.05 | (0.57, 1.94) | 1.000 |
| CI: confidence interval; OR: odds ratio; RBD: Rapid eye movement sleep behavior disorder; SE, standard error  a Hoehn-Yahr staging was taken as adjustment  b *p* value after Bonferroni correction  Bond fonts: *p* < 0.05 | | | | | | | | | | | | |

**Supplementary Table 5** **The association between PD rating scales and genotype of *GPNMB* rs156429 in male PD patients**

|  | *p* value | *p* value ^a^ | *p* value  (multiple pairwise-comparison between groups) | | |
| --- | --- | --- | --- | --- | --- |
|  | TT v.s. CT v.s. CC  (n = 171 v.s. 96 v.s. 13) | TT v.s. CT v.s. CC  (n = 171 v.s. 96 v.s. 13) | TT v.s. CT  (n = 171 v.s. 96) | TT v.s. CC  (n = 171 v.s. 13) | CT v.s. CC  (n = 96 v.s. 13) |
| SS-16 | 0.593 | 1.000 | 0.901 | 0.975 | 0.975 |
| HAMA | 0.502 | 1.000 | 0.709 | 0.435 | 0.435 |
| HAMD | 0.785 | 1.000 | 0.940 | 0.768 | 0.768 |
| BPI | 0.792 | 1.000 | 0.904 | 0.904 | 0.904 |
| RBD-HK | 0.692 | 1.000 | 0.864 | 0.692 | 0.692 |
| PDSS | 0.743 | 1.000 | 0.772 | 0.772 | 0.772 |
| PDQ39 | 0.274 | 1.000 | 0.385 | 0.385 | 0.385 |
| FSS | 0.748 | 1.000 | 0.748 | 0.748 | 0.748 |
| ESS | 0.848 | 1.000 | 0.781 | 0.781 | 0.781 |
| MDS-UPDRS | 0.700 | 1.000 | 0.600 | 0.600 | 0.600 |
| PART I | 0.635 | 1.000 | 0.704 | 0.704 | 0.704 |
| PART II | 0.681 | 1.000 | 0.786 | 0.713 | 0.713 |
| PART III | 0.957 | 1.000 | 0.927 | 0.927 | 0.927 |
| NMSS | 0.530 | 1.000 | 0.575 | 0.575 | 0.575 |
| cardiovascular | 0.681 | 1.000 | 0.605 | 0.605 | 0.605 |
| sleep | 0.198 | 1.000 | 0.271 | 0.271 | 0.421 |
| mood disorder | 0.563 | 1.000 | 0.739 | 0.523 | 0.523 |
| delusion | 0.578 | 1.000 | 0.850 | 0.949 | 0.850 |
| attention | 0.393 | 1.000 | 0.734 | 0.315 | 0.315 |
| gastrointestinal | 0.720 | 1.000 | 0.633 | 0.633 | 0.633 |
| urinary | 0.268 | 1.000 | 0.330 | 0.330 | 0.330 |
| sexual dysfunction | 0.920 | 1.000 | 0.994 | 0.994 | 0.994 |
| others | 0.413 | 1.000 | 0.809 | 0.333 | 0.333 |
| SCOPA-AUT | 0.819 | 1.000 | 0.705 | 0.705 | 0.705 |
| gastrointestinal | 0.360 | 1.000 | 0.921 | 0.261 | 0.261 |
| urinary | 0.455 | 1.000 | 0.699 | 0.454 | 0.454 |
| cardiovascular | 0.641 | 1.000 | 0.726 | 0.619 | 0.619 |
| skin | 0.388 | 1.000 | 0.531 | 0.399 | 0.399 |
| sexual dysfunction | 0.815 | 1.000 | 0.692 | 0.692 | 0.692 |
| drug usage | 0.729 | 1.000 | 0.789 | 0.789 | 0.789 |
| MMSE | 0.594 | 1.000 | 0.935 | 0.968 | 0.968 |
| MoCA | 0.595 | 1.000 | 0.854 | 0.854 | 0.854 |
| BPI, brief pain inventory; ESS, Epworth Sleepiness Scale; FSS, Fatigue severity scale; HAMA, Hamilton anxiety rating scale; HAMD, Hamilton depression rating scale; MDS, movement disorders society; MMSE, Mini-Mental State Examination; MoCA, Montreal Cognitive Assessment; NMSS, Non-Motor Symptoms Scale; PDQ-39, 39-item Parkinson's Disease Questionnaire; PDSS, Parkinson's disease sleep scale; RBD-HK, rapid eye movement sleep behavior disorder questionnaire-Hong Kong version; SCOPA-AUT, Scales for Outcomes in Parkinson's Disease-Autonomic questionnaire; SS-16, Sniffin' Sticks 16; UPDRS, Unified Parkinson's Disease Rating Scale  a *p* value after Bonferroni correction | | | | | |

**Supplementary Table 6** **The association between PD symptoms and genetic models of *GPNMB* rs156429 in male PD patients**

|  | Additive model | | | | | | Additive model (adjusted) ^a^ | | | | | |
| --- | --- | --- | --- | --- | --- | --- | --- | --- | --- | --- | --- | --- |
|  | beta | SE | *p* value | OR | 95% CI | *p* value ^b^ | beta | SE | *p* value | OR | 95% CI | *p* value ^b^ |
| dysphagia | 0.065 | 0.279 | 0.815 | 1.07 | (0.61, 1.82) | 1.000 | 0.204 | 0.305 | 0.503 | 1.23 | (0.66, 2.20) | 1.000 |
| sialorrhea | -0.139 | 0.217 | 0.521 | 0.87 | (0.56, 1.32) | 1.000 | -0.013 | 0.227 | 0.955 | 0.99 | (0.63, 1.53) | 1.000 |
| symptom “full very quickly” | 0.195 | 0.382 | 0.609 | 1.22 | (0.55, 2.50) | 1.000 | 0.205 | 0.400 | 0.609 | 1.23 | (0.53, 2.62) | 1.000 |
| constipation | -0.381 | 0.224 | 0.089 | 0.68 | (0.44, 1.05) | 1.000 | -0.344 | 0.234 | 0.142 | 0.71 | (0.44, 1.11) | 1.000 |
| nocturia | -0.397 | 0.209 | 0.057 | 0.67 | (0.44, 1.01) | 1.000 | -0.376 | 0.225 | 0.094 | 0.69 | (0.44, 1.06) | 1.000 |
| postural hypotension | -0.036 | 0.277 | 0.895 | 0.96 | (0.55, 1.63) | 1.000 | -0.001 | 0.284 | 0.998 | 1.00 | (0.56, 1.72) | 1.000 |
| daytime sweatiness | -0.052 | 0.231 | 0.821 | 0.95 | (0.60, 1.48) | 1.000 | -0.034 | 0.237 | 0.886 | 0.97 | (0.60, 1.53) | 1.000 |
| nocturnal sweatiness | -0.162 | 0.275 | 0.555 | 0.85 | (0.49, 1.43) | 1.000 | -0.132 | 0.279 | 0.635 | 0.88 | (0.50, 1.49) | 1.000 |
| light sensitivity | -1.918 | 1.030 | 0.063 | 0.15 | (0.01, 0.71) | 1.000 | -1.959 | 1.038 | 0.059 | 0.14 | (0.01, 0.70) | 1.000 |
| susceptible to cold | -0.060 | 0.288 | 0.834 | 0.94 | (0.52, 1.63) | 1.000 | -0.022 | 0.291 | 0.939 | 0.98 | (0.54, 1.70) | 1.000 |
| susceptible to heat | -0.051 | 0.303 | 0.867 | 0.95 | (0.51, 1.69) | 1.000 | 0.032 | 0.308 | 0.916 | 1.03 | (0.55, 1.86) | 1.000 |
| sexual dysfunction | -0.008 | 0.333 | 0.981 | 0.99 | (0.50, 1.86) | 1.000 | 0.044 | 0.335 | 0.895 | 1.05 | (0.52, 1.97) | 1.000 |
| hallucination | -0.154 | 0.435 | 0.724 | 0.86 | (0.34, 1.91) | 1.000 | -0.001 | 0.498 | 0.998 | 1.00 | (0.34, 2.51) | 1.000 |
| apathy | -0.106 | 0.265 | 0.691 | 0.90 | (0.52, 1.49) | 1.000 | -0.013 | 0.271 | 0.961 | 0.99 | (0.57, 1.66) | 1.000 |
| pain | 0.125 | 0.210 | 0.552 | 1.13 | (0.75, 1.71) | 1.000 | 0.176 | 0.219 | 0.421 | 1.19 | (0.77, 1.83) | 1.000 |
| urination disorders  (not nocturia) | -0.105 | 0.214 | 0.623 | 0.90 | (0.59, 1.36) | 1.000 | -0.050 | 0.225 | 0.825 | 0.95 | (0.61, 1.48) | 1.000 |
| fatigue | 0.071 | 0.206 | 0.728 | 1.07 | (0.72, 1.61) | 1.000 | 0.122 | 0.218 | 0.577 | 1.13 | (0.74, 1.74) | 1.000 |
| freezing of gait | -0.046 | 0.266 | 0.862 | 0.95 | (0.56, 1.59) | 1.000 | 0.033 | 0.284 | 0.907 | 1.03 | (0.58, 1.78) | 1.000 |
| tremor | -0.399 | 0.208 | 0.056 | 0.67 | (0.44, 1.01) | 1.000 | -0.335 | 0.221 | 0.128 | 0.72 | (0.46, 1.10) | 1.000 |
| hyposmia | 0.012 | 0.240 | 0.959 | 1.01 | (0.64, 1.64) | 1.000 | -0.013 | 0.245 | 0.956 | 0.99 | (0.61, 1.61) | 1.000 |
| probable RBD | -0.068 | 0.237 | 0.773 | 0.93 | (0.58, 1.47) | 1.000 | -0.010 | 0.242 | 0.967 | 0.99 | (0.61, 1.58) | 1.000 |
|  | Dominant model | | | | | | Dominant model (adjusted) ^a^ | | | | | |
|  | beta | SE | *p* value | OR | 95% CI | *p* value ^b^ | beta | SE | *p* value | OR | 95% CI | *p* value ^b^ |
| dysphagia | 0.098 | 0.335 | 0.769 | 1.10 | (0.57, 2.11) | 1.000 | 0.234 | 0.369 | 0.527 | 1.26 | (0.61, 2.60) | 1.000 |
| sialorrhea | -0.086 | 0.256 | 0.737 | 0.92 | (0.55, 1.51) | 1.000 | 0.076 | 0.270 | 0.779 | 1.08 | (0.63, 1.83) | 1.000 |
| symptom “full very quickly” | 0.269 | 0.467 | 0.564 | 1.31 | (0.51, 3.27) | 1.000 | 0.241 | 0.484 | 0.618 | 1.27 | (0.48, 3.28) | 1.000 |
| constipation | -0.351 | 0.259 | 0.175 | 0.70 | (0.42, 1.16) | 1.000 | -0.305 | 0.274 | 0.265 | 0.74 | (0.43, 1.26) | 1.000 |
| nocturia | -0.341 | 0.246 | 0.165 | 0.71 | (0.44, 1.15) | 1.000 | -0.317 | 0.267 | 0.234 | 0.73 | (0.43, 1.23) | 1.000 |
| postural hypotension | 0.075 | 0.326 | 0.818 | 1.08 | (0.56, 2.03) | 1.000 | 0.116 | 0.336 | 0.729 | 1.12 | (0.58, 2.16) | 1.000 |
| daytime sweatiness | 0.047 | 0.273 | 0.862 | 1.05 | (0.61, 1.78) | 1.000 | 0.073 | 0.282 | 0.796 | 1.08 | (0.62, 1.86) | 1.000 |
| nocturnal sweatiness | -0.295 | 0.326 | 0.366 | 0.74 | (0.39, 1.39) | 1.000 | -0.263 | 0.332 | 0.428 | 0.77 | (0.39, 1.46) | 1.000 |
| light sensitivity | -2.005 | 1.052 | 0.057 | 0.13 | (0.01, 0.71) | 1.000 | -2.041 | 1.057 | 0.053 | 0.13 | (0.01, 0.69) | 1.000 |
| susceptible to cold | 0.144 | 0.337 | 0.668 | 1.16 | (0.59, 2.23) | 1.000 | 0.203 | 0.343 | 0.554 | 1.22 | (0.62, 2.39) | 1.000 |
| susceptible to heat | 0.153 | 0.354 | 0.666 | 1.17 | (0.57, 2.32) | 1.000 | 0.267 | 0.365 | 0.464 | 1.31 | (0.63, 2.66) | 1.000 |
| sexual dysfunction | -0.108 | 0.400 | 0.788 | 0.90 | (0.40, 1.94) | 1.000 | -0.037 | 0.407 | 0.927 | 0.96 | (0.42, 2.11) | 1.000 |
| hallucination | -0.259 | 0.516 | 0.616 | 0.77 | (0.26, 2.05) | 1.000 | -0.168 | 0.591 | 0.777 | 0.85 | (0.25, 2.62) | 1.000 |
| apathy | -0.099 | 0.313 | 0.751 | 0.91 | (0.48, 1.66) | 1.000 | 0.015 | 0.323 | 0.964 | 1.01 | (0.53, 1.90) | 1.000 |
| pain | 0.174 | 0.252 | 0.490 | 1.19 | (0.73, 1.95) | 1.000 | 0.235 | 0.263 | 0.371 | 1.27 | (0.75, 2.12) | 1.000 |
| urination disorders  (not nocturia) | -0.096 | 0.254 | 0.706 | 0.91 | (0.55, 1.49) | 1.000 | -0.037 | 0.269 | 0.890 | 0.96 | (0.57, 1.63) | 1.000 |
| fatigue | 0.140 | 0.245 | 0.568 | 1.15 | (0.71, 1.86) | 1.000 | 0.208 | 0.261 | 0.425 | 1.23 | (0.74, 2.06) | 1.000 |
| freezing of gait | -0.024 | 0.316 | 0.939 | 0.98 | (0.52, 1.80) | 1.000 | 0.053 | 0.338 | 0.874 | 1.05 | (0.54, 2.04) | 1.000 |
| tremor | -0.386 | 0.246 | 0.117 | 0.68 | (0.42, 1.10) | 1.000 | -0.283 | 0.264 | 0.283 | 0.75 | (0.45, 1.26) | 1.000 |
| hyposmia | 0.097 | 0.285 | 0.735 | 1.10 | (0.63, 1.94) | 1.000 | 0.053 | 0.292 | 0.857 | 1.05 | (0.60, 1.88) | 1.000 |
| probable RBD | 0.010 | 0.281 | 0.971 | 1.01 | (0.58, 1.74) | 1.000 | 0.091 | 0.289 | 0.754 | 1.09 | (0.62, 1.92) | 1.000 |
|  | Recessive model | | | | | | Recessive model (adjusted) ^a^ | | | | | |
|  | beta | SE | *p* value | OR | 95% CI | *p* value ^b^ | beta | SE | *p* value | OR | 95% CI | *p* value ^b^ |
| dysphagia | -0.026 | 0.787 | 0.973 | 0.97 | (0.15, 3.80) | 1.000 | 0.309 | 0.811 | 0.703 | 1.36 | (0.20, 5.67) | 1.000 |
| sialorrhea | -0.659 | 0.670 | 0.326 | 0.52 | (0.11, 1.74) | 1.000 | -0.543 | 0.685 | 0.428 | 0.58 | (0.13, 2.03) | 1.000 |
| symptom “full very quickly” | 0.084 | 1.068 | 0.937 | 1.09 | (0.06, 5.99) | 1.000 | 0.273 | 1.084 | 0.801 | 1.31 | (0.07, 7.62) | 1.000 |
| constipation | -1.176 | 0.779 | 0.131 | 0.31 | (0.05, 1.18) | 1.000 | -1.126 | 0.791 | 0.155 | 0.32 | (0.05, 1.28) | 1.000 |
| nocturia | -1.301 | 0.670 | 0.052 | 0.27 | (0.06, 0.91) | 1.000 | -1.234 | 0.689 | 0.073 | 0.29 | (0.06, 1.03) | 1.000 |
| postural hypotension | -0.915 | 1.053 | 0.385 | 0.40 | (0.02, 2.11) | 1.000 | -0.846 | 1.060 | 0.425 | 0.43 | (0.02, 2.31) | 1.000 |
| daytime sweatiness | -0.783 | 0.781 | 0.316 | 0.46 | (0.07, 1.75) | 1.000 | -0.764 | 0.788 | 0.332 | 0.47 | (0.07, 1.82) | 1.000 |
| nocturnal sweatiness | 0.314 | 0.677 | 0.643 | 1.37 | (0.30, 4.67) | 1.000 | 0.360 | 0.688 | 0.601 | 1.43 | (0.31, 5.05) | 1.000 |
| light sensitivity | -- | -- | 0.993 | -- | -- | 1.000 | -- | -- | 0.993 | -- | -- | 1.000 |
| susceptible to cold | -- | -- | 0.988 | -- | -- | 1.000 | -- | -- | 0.989 | -- | -- | 1.000 |
| susceptible to heat | -- | -- | 0.989 | -- | -- | 1.000 | -- | -- | 0.989 | -- | -- | 1.000 |
| sexual dysfunction | 0.440 | 0.794 | 0.580 | 1.55 | (0.23, 6.17) | 1.000 | 0.455 | 0.802 | 0.570 | 1.58 | (0.23, 6.41) | 1.000 |
| hallucination | 0.203 | 1.071 | 0.849 | 1.23 | (0.07, 6.81) | 1.000 | 0.770 | 1.112 | 0.488 | 2.16 | (0.11, 13.65) | 1.000 |
| apathy | -0.285 | 0.784 | 0.716 | 0.75 | (0.11, 2.91) | 1.000 | -0.191 | 0.793 | 0.810 | 0.83 | (0.12, 3.28) | 1.000 |
| pain | 0.027 | 0.584 | 0.963 | 1.03 | (0.30, 3.16) | 1.000 | 0.098 | 0.602 | 0.871 | 1.10 | (0.32, 3.57) | 1.000 |
| urination disorders  (not nocturia) | -0.298 | 0.614 | 0.627 | 0.74 | (0.20, 2.34) | 1.000 | -0.183 | 0.632 | 0.772 | 0.83 | (0.22, 2.76) | 1.000 |
| fatigue | -0.207 | 0.570 | 0.717 | 0.81 | (0.26, 2.51) | 1.000 | -0.173 | 0.593 | 0.771 | 0.84 | (0.26, 2.77) | 1.000 |
| freezing of gait | -0.237 | 0.785 | 0.763 | 0.79 | (0.12, 3.06) | 1.000 | -0.038 | 0.806 | 0.962 | 0.96 | (0.14, 3.95) | 1.000 |
| tremor | -0.999 | 0.613 | 0.103 | 0.37 | (0.10, 1.16) | 1.000 | -1.057 | 0.633 | 0.095 | 0.35 | (0.09, 1.15) | 1.000 |
| hyposmia | -0.416 | 0.629 | 0.509 | 0.66 | (0.20, 2.54) | 1.000 | -0.371 | 0.646 | 0.566 | 0.69 | (0.20, 2.72) | 1.000 |
| probable RBD | -0.675 | 0.781 | 0.387 | 0.51 | (0.08, 1.96) | 1.000 | -0.649 | 0.789 | 0.411 | 0.52 | (0.08, 2.06) | 1.000 |
|  | Overdominant model | | | | | | Overdominant model (adjusted) ^a^ | | | | | |
|  | beta | SE | *p* value | OR | 95% CI | *p* value ^b^ | beta | SE | *p* value | OR | 95% CI | *p* value ^b^ |
| dysphagia | -0.108 | 0.342 | 0.752 | 0.90 | (0.46, 1.79) | 1.000 | -0.183 | 0.377 | 0.627 | 0.83 | (0.40, 1.78) | 1.000 |
| sialorrhea | -0.026 | 0.262 | 0.922 | 0.97 | (0.58, 1.64) | 1.000 | -0.179 | 0.277 | 0.518 | 0.84 | (0.49, 1.44) | 1.000 |
| symptom “full very quickly” | -0.265 | 0.475 | 0.577 | 0.77 | (0.31, 2.02) | 1.000 | -0.201 | 0.494 | 0.685 | 0.82 | (0.32, 2.25) | 1.000 |
| constipation | 0.182 | 0.265 | 0.491 | 1.20 | (0.72, 2.03) | 1.000 | 0.134 | 0.280 | 0.631 | 1.14 | (0.66, 1.99) | 1.000 |
| nocturia | 0.129 | 0.252 | 0.609 | 1.14 | (0.69, 1.87) | 1.000 | 0.101 | 0.274 | 0.712 | 1.11 | (0.65, 1.90) | 1.000 |
| postural hypotension | -0.210 | 0.331 | 0.526 | 0.81 | (0.43, 1.57) | 1.000 | -0.244 | 0.341 | 0.474 | 0.78 | (0.40, 1.55) | 1.000 |
| daytime sweatiness | -0.176 | 0.278 | 0.526 | 0.84 | (0.49, 1.46) | 1.000 | -0.204 | 0.288 | 0.478 | 0.82 | (0.47, 1.44) | 1.000 |
| nocturnal sweatiness | 0.388 | 0.343 | 0.257 | 1.47 | (0.77, 2.97) | 1.000 | 0.365 | 0.349 | 0.295 | 1.44 | (0.74, 2.93) | 1.000 |
| light sensitivity | 1.798 | 1.052 | 0.087 | 6.04 | (1.15, 111.26) | 1.000 | 1.853 | 1.060 | 0.080 | 6.38 | (1.19, 118.44) | 1.000 |
| susceptible to cold | -0.384 | 0.339 | 0.257 | 0.68 | (0.35, 1.34) | 1.000 | -0.450 | 0.345 | 0.192 | 0.64 | (0.32, 1.27) | 1.000 |
| susceptible to heat | -0.387 | 0.356 | 0.277 | 0.68 | (0.34, 1.38) | 1.000 | -0.503 | 0.367 | 0.171 | 0.60 | (0.30, 1.26) | 1.000 |
| sexual dysfunction | 0.219 | 0.420 | 0.601 | 1.25 | (0.56, 2.97) | 1.000 | 0.150 | 0.426 | 0.725 | 1.16 | (0.52, 2.80) | 1.000 |
| hallucination | 0.325 | 0.542 | 0.549 | 1.38 | (0.50, 4.42) | 1.000 | 0.362 | 0.628 | 0.565 | 1.44 | (0.45, 5.56) | 1.000 |
| apathy | 0.053 | 0.321 | 0.870 | 1.05 | (0.57, 2.01) | 1.000 | -0.051 | 0.331 | 0.878 | 0.95 | (0.50, 1.85) | 1.000 |
| pain | -0.178 | 0.258 | 0.491 | 0.84 | (0.51, 1.39) | 1.000 | -0.229 | 0.270 | 0.397 | 0.80 | (0.47, 1.35) | 1.000 |
| urination disorders  (not nocturia) | 0.045 | 0.261 | 0.864 | 1.05 | (0.63, 1.75) | 1.000 | 0.004 | 0.277 | 0.990 | 1.00 | (0.58, 1.73) | 1.000 |
| fatigue | -0.189 | 0.252 | 0.454 | 0.83 | (0.50, 1.36) | 1.000 | -0.257 | 0.270 | 0.341 | 0.77 | (0.45, 1.31) | 1.000 |
| freezing of gait | -0.018 | 0.323 | 0.956 | 0.98 | (0.53, 1.88) | 1.000 | -0.063 | 0.346 | 0.855 | 0.94 | (0.48, 1.88) | 1.000 |
| tremor | 0.218 | 0.252 | 0.387 | 1.24 | (0.76, 2.04) | 1.000 | 0.088 | 0.271 | 0.745 | 1.09 | (0.64, 1.86) | 1.000 |
| hyposmia | -0.186 | 0.295 | 0.528 | 0.83 | (0.46, 1.47) | 1.000 | -0.132 | 0.303 | 0.662 | 0.88 | (0.48, 1.57) | 1.000 |
| probable RBD | -0.123 | 0.286 | 0.668 | 0.88 | (0.51, 1.56) | 1.000 | -0.205 | 0.295 | 0.487 | 0.81 | (0.46, 1.46) | 1.000 |
| CI: confidence interval; OR: odds ratio; RBD: Rapid eye movement sleep behavior disorder; SE, standard error  a Hoehn-Yahr staging was taken as adjustment  b *p* value after Bonferroni correction | | | | | | | | | | | | |
